# The QChip1 knowledgebase and microarray for precision medicine in Qatar

**DOI:** 10.1038/s41525-021-00270-0

**Published:** 2022-01-19

**Authors:** Juan L. Rodriguez-Flores, Radja Messai-Badji, Amal Robay, Ramzi Temanni, Najeeb Syed, Monika Markovic, Eiman Al-khayat, Fatima Qafoud, Zafar Nawaz, Ramin Badii, Yasser Al-Sarraj, Hamdi Mbarek, Wadha Al-Muftah, Muhammad Alvi, Mahboubeh R. Rostami, Juan Carlos Martinez Cruzado, Jason G. Mezey, Alya Al Shakaki, Joel A. Malek, Matthew B. Greenblatt, Khalid A. Fakhro, Khaled Machaca, Ajayeb Al-Nabet, Nahla Afifi, Andrew Brooks, Said I. Ismail, Asmaa Althani, Ronald G. Crystal

**Affiliations:** 1 Department of Genetic Medicine, Weill Cornell Medicine, New York, NY USA; 2 Qatar Genome Program, Qatar Foundation, Doha, Qatar; 3 Weill Cornell Medicine, Doha, Qatar; 4 Department of Human Genetics, Sidra Medicine, Doha, Qatar; 5 Qatar Biobank for Medical Research, Qatar Foundation, Doha, Qatar; 6 Diagnostic Genomic Division, Hamad Medical Corporation, Doha, Qatar; 7 Department of Biology, University of Puerto Rico at Mayagüez, Mayagüez Puerto Rico, USA; 8 Department of Computational Biology, Cornell University, Ithaca, NY USA; 9 Department of Pathology and Laboratory Medicine, Weill Cornell Medicine, New York, NY USA; 10 RUCDR Infinite Biologics, Piscataway, NJ USA; 11 Department of Genetics, Rutgers University, New Brunswick, NJ USA; 12 Biomedical Research Center, Qatar University, Doha, Qatar; 13 Present Address: Regeneron Genetics Center, Tarrytown, NY USA

**Keywords:** Medical research, Risk factors

## Abstract

Risk genes for Mendelian (single-gene) disorders (SGDs) are consistent across populations, but pathogenic risk variants that cause SGDs are typically population-private. The goal was to develop “QChip1,” an inexpensive genotyping microarray to comprehensively screen newborns, couples, and patients for SGD risk variants in Qatar, a small nation on the Arabian Peninsula with a high degree of consanguinity. Over 10^8^ variants in 8445 Qatari were identified for inclusion in a genotyping array containing 165,695 probes for 83,542 known and potentially pathogenic variants in 3438 SGDs. QChip1 had a concordance with whole-genome sequencing of 99.1%. Testing of QChip1 with 2707 Qatari genomes identified 32,674 risk variants, an average of 134 pathogenic alleles per Qatari genome. The most common pathogenic variants were those causing homocystinuria (1.12% risk allele frequency), and Stargardt disease (2.07%). The majority (85%) of Qatari SGD pathogenic variants were not present in Western populations such as European American, South Asian American, and African American in New York City and European and Afro-Caribbean in Puerto Rico; and only 50% were observed in a broad collection of data across the Greater Middle East including Kuwait, Iran, and United Arab Emirates. This study demonstrates the feasibility of developing accurate screening tools to identify SGD risk variants in understudied populations, and the need for ancestry-specific SGD screening tools.

## Introduction

A major goal of precision medicine is to optimize medical care for subgroups of patients based on genetic and/or molecular profiling^1^. A challenge in widespread adaptation of genetic profiling is the genome variability among different population groups^2^. One example is the identification of pathogenic variants in (Mendelian) single gene disorders (SGDs). While the same genes are responsible, there is considerable variability across populations in the specific causative pathogenic variants^3^. For example, while all pathogenic variants causing cystic fibrosis affect the *CFTR* gene, the common pathogenic variant observed in Puerto Rico^4^ is different from the variant observed in Qatar^5^ and both are different from the pathogenic variants common in European populations^6^. A recent analysis of ClinVar, the main NCBI database of pathogenic variants causative of SGDs, shows a significant bias towards pathogenic variants observed in European ancestry individuals^2^. As is the case for Hispanics, Blacks, and other non-European groups, SGD pathogenic variants found in Greater Middle Eastern populations are under-reported. Since screening technologies depend on public resources such as ClinVar^7^, OMIM^8^, and 1000 Genomes Project^9^ for source data, there are limited screening platforms to assess SGD pathogenic variants in the Greater Middle East^10^.

A striking example of this is the Qatari population^11,12^. The inhabitants of Qatar include approximately 300 thousand Qataris and 2.5 million expatriates^13^. The Qataris are comprised of distinct genetic subgroups^11,14^. The proportion of consanguineous marriage among Qataris is high^15^, leading to longer runs of homozygosity^16^. In addition, the tribal nature of marriages, where individuals select a mate from a limited gene pool that are members of the same tribe, contributes to higher chance of homozygosity for a pathogenic founder variant derived from a common ancestor, such as the well-known p.Arg366Cys CBS variant linked to homocystinuria^17^.

In prior studies, we and others have identified SGD pathogenic variants that are common in the Qatari population^3^ and in other Greater Middle East populations^18^, including many pathogenic variants that are only observed in Qatari genomes or are at an enriched (higher) risk allele frequency compared to populations outside of the Greater Middle East^14^. At present, there is a limited screening of the Qatari populations for inherited pathogenic variants^19^.

The focus of this study is to develop “QChip1,” a genotyping microarray designed as a research and screening tool capable of enabling precision medicine of Qataris. The aim for QChip1 was to enable accurate and comprehensive screening for SGD pathogenic variants in Qatari newborns, premarital couples and patients presenting to the clinic. First, we analyzed genetic data from 8445 Qataris, including whole-genome sequence (WGS), whole-exome sequence (WES), and clinical pathology case reports from affected families. Using these data, a Qatari Genome Knowledgebase was constructed, containing known and predicted pathogenic variants in SGDs. Second, with this knowledgebase, QChip1 was designed to assess the Qatari genome for SGD pathogenic variants in the knowledgebase. Third, QChip1 accuracy was confirmed by comparison of QChip1 genotypes to WGS data for a batch of Qatari genomes. Fourth, genomes from Qataris and residents of New York City (NYC), and Puerto Rico (PR) were genotyped on QChip1 to determine the prevalence of SGD pathogenic variants in Qataris and to compare this to other populations. The analysis demonstrated that QChip1 is highly accurate in identifying deleterious variants in Qataris, and that the majority of pathogenic variants among Qataris are Qatari-specific or Qatari-enriched. Overall, this study demonstrates the value of a custom genotyping array for precision medicine identification of pathogenic variants that cause single-gene disorders in human populations absent from or underrepresented by common knowledgebases used for pathogenic variant screening assay design^7–9,20,21^. In the interest of the advancement of science and open data sharing, a list of variants on the array, the genes and disorders with a known or potential link to the variants, and the prevalence of these variants in Qatar, Kuwait, NYC, and PR will be made available to the public through the QChip Browser (http://qchip.biohpc.cornell.edu), as well as through our 3rd party data sharing repositories at FigShare (https://figshare.com/projects/QChip1/120108) and NCBI BioProject (https://www.ncbi.nlm.nih.gov/bioproject/PRJNA774497).

## Results

### Construction of the Qatari Genome Knowledgebase

The Qatari Genome Knowledgebase of single gene coding sequence pathogenic and potentially pathogenic variants was based on sequence data from 8416 Qataris, including 6218 whole-genome sequence of Qataris recruited by the Qatar BioBank (QBB)^22,23^ and sequenced by the Qatar Genome Program (QGP)^24,25^, 180 whole-genome sequences^12,26^ and 1297 exome sequences^11^ of Qataris recruited by Weill Cornell Medicine Qatar and sequenced by Illumina, Beijing Genomics Institute (BGI) or the New York Genome Center (NYGC), and 721 clinical reports from Hamad Medical Corporation (Supplementary Table 1). After filtering to remove variants observed in multiple cohorts, the analysis yielded 104,473,390 total variants in 20,069 genes in the Qatari population, including 87,813,560 single nucleotide variants (SNV) and 16,659,829 indels (Table 1); below we refer to this dataset as the Qatar Genome Knowlegebase (QGK). Assessment of QGK for ClinVar pathogenic variants and genes yielded a list of 10,490,820 variants in 3770 genes known to ClinVar. Parallel assessment of QGK for moderate or high impact variants in protein coding genes using SnpEff identified 805,649 variants in 19,770 genes (Table 1, Supplementary Table 2). The SnpEff list of moderate/high impact predicted variants was intersected with the ClinVar list of known variants and known genes to generate a final list of 207,370 pathogenic variants in 3770 genes, including 196,855 single nucleotide variants (SNVs) in 3769 genes and 10,515 indels in 1897 genes. This final list of variants included 13,891 (7%) predicted high impact (e.g., nonsense, frame shift and other loss of function) and 193,479 (93%) predicted moderate impact (e.g., missense variants).

**Table 1:** Step 1: Identification of pathogenic variants and genes in the Qatari Genome.

Category	Identification of variants/genes	Variants (n)	Genes (n)
All variants/genes^a^	Qatari Genome Program	94,852,664	19,965
	Weill Cornell Medicine exomes	767,957	19,385
	Weill Cornell Medicine genomes	28,331,826	18,499
	Hamad Medical Corporation	727	513
Comprehensive list	All Qatari variants/genes	104,473,390	20,069
	Single nucleotide variants	87,813,560	20,042
	Indels	16,659,829	19,898
Variants of interest for SGD research and screening	ClinVar variants/genes, including pathogenic and non-pathogenic	10,490,820	3770
	SnpEff computationally predicted pathogenic variants/genes for research	805,649	19,770
Comprehensive list^b^	Qatari variants/genes of interest for SDG research and screening	207,370	3770
	Single nucleotide variants	196,855	3769
	Indels	10,515	1897

^a^A list of all Qatari variants and genes was compiled from all Qatari variants and the genes responsible for these variants identified in datasets described in Supplementary Table 1.

^b^The comprehensive list of all Qatari variants of interest for research and screening in single gene (Mendelian) disorder (SGD) was compiled from the subset of the list of all Qatari variants/genes identified in ClinVar and predicted to be of high or moderate impact by SnpEff.

### Design of QChip1

For each variant in the Axiom QChip design, one or more probesets were added to the design, depending on the computationally predicted difficulty of obtaining a high-quality genotype, the priority of the variant, and available space on the array. QChip0 consisted of a total of 184,713 probes organized in 159,377 probesets for genotyping 91,942 variants in 3540 genes (Table 2). The additional probesets represent variants not previously genotyped by Thermo Fisher (formerly Affymetrix) arrays, for these novel variants (67,435 or 73.3% of 91,942) 2 or more probes were included in the probeset, while for known variants (24,507 or 26.7%) a single probe was included in the probeset.

**Table 2:** Step 2: Design of QChip1 based on the predicted pathogenic variants in the Qatari Genome.

Microarray^a^	Probes (*n*)	Variant sites (*n*)	Genes (*n*)
QChip0	184,713	91,942	3540
SNV	179,257	89,696	3529
Indels	5456	2246	10,665
QChip1	70,715	61,592	3438
SNV	69,745	60,858	3472
Indels	970	734	491

^a^Based on the comprehensive list of Qatari variants and genes of interest for SGD research and screening (Table 1), QChip0, the precursor of QChip1, was designed on the Axion platform with 184,713 probes representing 91,942 variants and 3540 genes (see Methods for a description of prioritization of variants/genes and choice of probes). QChip0 was tested with *n* = 26 Qatari DNA samples for which whole-genome sequencing was available. The poorly performing probes with low-quality genotype sites were eliminated, resulting in the final design of QChip1 with 70,715 probes representing 61,592 variants and 3438 genes.

QChip0 was then tested on 26 Qatari genomes for which WGS was available. Concordance was 99.7% ± 0.002 for *n* = 61,592 of *n* = 91,942 variant sites with non-missing genotypes in both WGS and QChip0 for all *n* = 26 samples. This high-confidence dataset consisted of 70,715 probes in 61,592 probesets for genotyping of 61,592 variants in 3438 genes (61,195 SNV probesets for 61,195 variants in 3476 genes, and 397 indel probesets for 397 variants in 300 genes), resulting in the final design of QChip1 (Table 2). Of these probes, 61,565 were autosomal and a small proportion (*n* = 27; 0.04%) non-autosomal (located in ChrX, ChrY, or MtDNA).

### Testing of QChip1

The single nucleotide variants and indels represented on QChip1 were tested with an additional 473 Qatari genomes for which whole-genome sequencing was available^24^. After selection of the top performing probeset for each variant, probesets that were consistently top-performing across batches were compared to WGS genotypes. A total of 27,850 ± 0.75 variant sites where a high-confidence genotype was obtained for both QChip and WGS were compared, concordance was 99.1% ± 0.00034 (Table 3). Concordance was high for indels (92.4% ± 0.0057) and SNVs (99.2% ± 0.00034).

**Table 3:** Step 3: Concordance of QChip1 compared to whole-genome sequencing^a^.

Population^b^		Indel				SNV				Indel + SNV			
		Concordant	Discordant	Total	Concordance rate	Concordant	Discordant	Total	Concordance rate	Concordant	Discordant	Total	Concordance rate
Qatari	Average	1.7 × 10^1^	1.4 × 10^0^	1.8 × 10^1^	9.2 × 10^−1^	2.8 × 10^4^	2.2 × 10^2^	2.8 × 10^4^	9.9 × 10^−1^	2.8 × 10^4^	2.3 × 10^2^	2.8 × 10^4^	9.9 × 10^−1^
	Standard deviation	2.5 × 10^0^	1.2 × 10^0^	2.7 × 10^0^	6.4 × 10^−2^	1.1 × 10^2^	1.1 × 10^2^	8.9 × 10^0^	3.8 × 10^−3^	1.1 × 10^2^	1.1 × 10^2^	8.3 × 10^0^	3.8 × 10^−3^
	Sample size	4.7 × 10^2^	4.7 × 10^2^	4.7 × 10^2^	4.7 × 10^2^	4.7 × 10^2^	4.7 × 10^2^	4.7 × 10^2^	4.7 × 10^2^	4.7 × 10^2^	4.7 × 10^2^	4.7 × 10^2^	4.7 × 10^2^
	Confidence interval	2.4 × 10^−1^	1.1 × 10^−1^	2.5 × 10^−1^	6.0 × 10^−3^	1.0 × 10^1^	9.9 × 10^0^	8.4 × 10^−1^	3.6 × 10^−4^	1.0 × 10^1^	1.0 × 10^1^	7.8 × 10^−1^	3.6 × 10^−4^
QGP_PAR	Average	1.6 × 10^1^	1.5 × 10^0^	1.9 × 10^1^	9.2 × 10^−1^	2.8 × 10^4^	2.2 × 10^2^	2.8 × 10^4^	9.9 × 10^−1^	2.8 × 10^4^	2.2 × 10^2^	2.8 × 10^4^	9.9 × 10^−1^
	Standard deviation	2.4 × 10^0^	1.1 × 10^0^	2.3 × 10^0^	5.6 × 10^−2^	8.6 × 10^1^	8.5 × 10^1^	2.3 × 10^0^	3.1 × 10^−3^	8.6 × 10^1^	8.6 × 10^1^	0.0 × 10^0^	3.1 × 10^−3^
	Sample size	1.4 × 10^2^	1.5 × 10^2^	1.5 × 10^2^	1.5 × 10^2^	1.5 × 10^2^	1.5 × 10^2^	1.5 × 10^2^	1.5 × 10^2^	1.5 × 10^2^	1.5 × 10^2^	1.5 × 10^2^	1.5 × 10^2^
	Confidence interval	4.3 × 10^−1^	1.9 × 10^−1^	3.9 × 10^−1^	9.4 × 10^−3^	1.4 × 10^1^	1.4 × 10^1^	3.9 × 10^−1^	5.1 × 10^−4^	1.4 × 10^1^	1.4 × 10^1^	Undefined^3^	5.2 × 10^−4^
QGP_GAR	Average	1.7 × 10^1^	1.5 × 10^0^	1.9 × 10^1^	9.2 × 10^−1^	2.8 × 10^4^	2.2 × 10^2^	2.8 × 10^4^	9.9 × 10^−1^	2.8 × 10^4^	2.2 × 10^2^	2.8 × 10^4^	9.9 × 10^−1^
	Standard deviation	2.1 × 10^0^	1.1 × 10^0^	2.3 × 10^0^	5.6 × 10^−2^	8.6 × 10^1^	8.5 × 10^1^	2.3 × 10^0^	3.1 × 10^−3^	8.6 × 10^1^	8.6 × 10^1^	0.0 × 10^0^	3.1 × 10^−3^
	Sample size	1.5 × 10^2^	1.5 × 10^2^	1.5 × 10^2^	1.5 × 10^2^	1.5 × 10^2^	1.5 × 10^2^	1.5 × 10^2^	1.5 × 10^2^	1.5 × 10^2^	1.5 × 10^2^	1.5 × 10^2^	1.5 × 10^2^
	Confidence interval	3.5 × 10^−1^	1.9 × 10^−1^	3.9 × 10^−1^	9.4 × 10^−3^	1.4 × 10^1^	1.4 × 10^1^	3.9 × 10^−1^	5.1 × 10^−4^	1.4 × 10^1^	1.4 × 10^1^	Undefined^3^	5.2 × 10^−4^
QGP_ADM	Average	1.7 × 10^1^	1.4 × 10^0^	1.9 × 10^1^	9.3 × 10^−1^	2.8 × 10^4^	2.4 × 10^2^	2.8 × 10^4^	9.9 × 10^−1^	2.8 × 10^4^	2.4 × 10^2^	2.8 × 10^4^	9.9 × 10^−1^
	Standard deviation	2.6 × 10^0^	1.1 × 10^0^	2.8 × 10^0^	5.8 × 10^−2^	7.5 × 10^1^	7.4 × 10^1^	2.2 × 10^1^	2.7 × 10^−3^	7.5 × 10^1^	7.5 × 10^1^	2.1 × 10^1^	2.7 × 10^−3^
	Sample size	7.1 × 10^1^	7.1 × 10^1^	7.1 × 10^1^	7.1 × 10^1^	7.1 × 10^1^	7.1 × 10^1^	7.1 × 10^1^	7.1 × 10^1^	7.1 × 10^1^	7.1 × 10^1^	7.1 × 10^1^	7.1 × 10^1^
	Confidence interval	6.3 × 10^−1^	2.0 × 10^−1^	6.9 × 10^−1^	1.4 × 10^−2^	1.8 × 10^1^	1.8 × 10^1^	5.4 × 10^0^	6.5 × 10^−4^	1.8 × 10^1^	1.8 × 10^1^	5.2 × 10^0^	6.6 × 10^−4^
QGP_WEP	Average	1.7 × 10^1^	1.3 × 10^0^	1.8 × 10^1^	9.3 × 10^−1^	2.8 × 10^4^	2.1 × 10^2^	2.0 × 10^4^	9.9 × 10^−1^	2.8 × 10^4^	2.1 × 10^2^	2.8 × 10^4^	9.9 × 10^−1^
	Standard deviation	2.6 × 10^0^	1.2 × 10^0^	2.7 × 10^0^	6.5 × 10^−2^	6.2 × 10^1^	6.1 × 10^1^	2.7 × 10^0^	2.2 × 10^−3^	6.2 × 10^1^	6.2 × 10^1^	0.0 × 10^0^	2.2 × 10^−3^
	Sample size	8.8 × 10^1^	8.8 × 10^1^	8.8 × 10^1^	8.8 × 10^1^	8.8 × 10^1^	8.8 × 10^1^	8.8 × 10^1^	8.8 × 10^1^	8.8 × 10^1^	8.8 × 10^1^	8.8 × 10^1^	8.8 × 10^1^
	Confidence interval	5.7 × 10^−1^	2.7 × 10^−1^	6.0 × 10^−1^	1.4 × 10^−2^	1.4 × 10^1^	1.3 × 10^1^	6.0 × 10^−1^	4.8 × 10^−4^	1.4 × 10^1^	1.4 × 10^1^	Undefined^3^	4.9 × 10^−4^
QGP_SAS	Average	1.8 × 10^1^	0.0 × 10^0^	1.8 × 10^1^	1.0 × 10^0^	2.8 × 10^4^	1.4 × 10^2^	2.8 × 10^4^	9.9 × 10^−1^	2.8 × 10^4^	1.4 × 10^2^	2.8 × 10^4^	9.9 × 10^−1^
	Standard deviation	1.5 × 10^0^	0.0 × 10^0^	1.5 × 10^0^	0.0 × 10^0^	7.0 × 10^0^	5.5 × 10^0^	1.5 × 10^0^	2.0 × 10^−4^	5.5 × 10^0^	5.5 × 10^0^	0.0 × 10^0^	2.0 × 10^−4^
	Sample size	2.0 × 10^0^	2.0 × 10^0^	2.0 × 10^0^	2.0 × 10^0^	2.0 × 10^0^	2.0 × 10^0^	2.0 × 10^0^	2.0 × 10^0^	2.0 × 10^0^	2.0 × 10^0^	2.0 × 10^0^	2.0 × 10^0^
	Confidence interval	2.2 × 10^0^	Undefined^c^	2.2 × 10^0^	#NUM!	1.0 × 10^1^	8.0 × 10^0^	2.2 × 10^0^	2.9 × 10^−4^	8.0 × 10^0^	8.0 × 10^0^	Undefined^3^	2.9 × 10^−4^
QGP_AFR	Average	1.9 × 10^1^	1.4 × 10^0^	2.1 × 10^1^	9.4 × 10^−1^	2.8 × 10^4^	3.0 × 10^2^	2.8 × 10^4^	9.9 × 10^−1^	2.8 × 10^4^	3.0 × 10^2^	2.8 × 10^4^	9.9 × 10^−1^
	Standard deviation	2.8 × 10^0^	1.3 × 10^0^	2.9 × 10^0^	5.7 × 10^−2^	7.1 × 10^1^	7.0 × 10^1^	2.9 × 10^0^	2.5 × 10^−3^	7.1 × 10^1^	7.1 × 10^1^	0.0 × 10^0^	2.5 × 10^−3^
	Sample size	2.7 × 10^1^	2.7 × 10^1^	2.7 × 10^1^	2.7 × 10^1^	2.7 × 10^1^	2.7 × 10^1^	2.7 × 10^1^	2.7 × 10^1^	2.7 × 10^1^	2.7 × 10^1^	2.7 × 10^1^	2.7 × 10^1^
	Confidence interval	1.0 × 10^0^	4.9 × 10^−1^	1.2 × 10^0^	2.2 × 10^−2^	2.8 × 10^1^	2.8 × 10^1^	1.2 × 10^0^	9.9 × 10^−4^	2.8 × 10^1^	2.8 × 10^1^	Undefined^3^	1.0 × 10^−3^

^a^In order to assess the quality of QChip1 data, genotypes were generated for *n* = 473 Qataris for all QChip1 sites compared to whole-genome sequencing in data. Genotypes were generated for all sites, including both reference and variant genotypes in whole-genome sequencing. The concordance between QChip1 and whole-genome sequencing indels and single nucleotide variants (SNV) genotypes were compared. Shown for all and for each population and for each variant class (indel, SNV, both) the average, standard deviation, sample size, and 95% confidence interval for the number of concordant variants, the number of discordant variants, the total number of variants compared, and the concordance rate.

^b^Populations include: Qatari (all Qatari) and subpopulations: QGP_PAR (Peninsular Arabs); QGP_GAR (General Arabs); QGP_ADM (Admixed Arabs); QGP_WEP (Arabs of Wester Eurasia and Persia); QGP_SAS (South Asian Arabs); and QGP_AFR (African Arabs).

^c^Unable to calculate confidence interval when the standard deviation equals zero.

QChip1 was then used to determine the prevalence in the Qatari population and in non-Qatari populations for variants of interest for SGD pathogenicity research and screening in Qatar. Genotyping of *n* = 2708 Qatari, *n* = 226 European-American, South Asian American and African-American New York City (NYC) residents and *n* = 51 European and Afro-Caribbean Puerto Rico (PR) residents was conducted and analyzed as a single batch, including data from the first two (QChip0/QChip1) batches described above and a third batch with the rest of the samples. Probesets were again filtered based on performance, and variants were filtered based on missing genotype rate (<10%) low concordance with WGS in batches 1 or 2 (>90%) and minor allele frequency (<5%). The final set of variants for analysis included *n* = 32,674 SNVs. In order to assess the utility of QChip1 for use in other populations of the Greater Middle East (GME), the allele frequency of these variants was obtained for *n* = 540 Kuwaiti exomes and each variant was checked for presence in the Center for Arab Genetic Disorders (CAGS) database (http://cags.org.ae).

### Use of QChip1

Among the 2,708 Qatari genomes tested, QChip1 identified a median of 2 homozygotes and 130 heterozygotes for SNVs of interest for SGD pathogenicity research and screening (Table 4). When assessed by Qatari subpopulations^25^, the highest median number (*n* = 205) of SNVs were identified in the Peninsular Arab subpopulation, 1.6-fold greater than the average median for the General Arab (109), Arabs of Western Eurasia and Persia (132), South Asian Arabs (137) and African Arab (129) subpopulations.

**Table 4:** Step 4: Use QChip1 to assess average number of single nucleotide variants per genome of interest for SGD research and screening in Qataris and other populations^a^.

Genomes assessed (*n*)^b,c^	SNVs of interest for SGD research and screening identified by QChip1				Median number of identified SNVs
	Homozygous	Heterozygous	Wild-type	Missing	
Qatari (*n* = 2708)	2	130	32,501	37	134
QGP_PAR (*n* = 510)	2	107	32,530	33	109
QGP_GAR (*n* = 280)	2	203	32,418	43	205
QGP_WEP (*n* = 768)	1	131	32,502	38	132
QGP_SAS (*n* = 504)	1	136	32,494	38	137
QGP_AFR (*n* = 646)	1	128	32,504	35	129

^a^In order to compare the precision medicine value of QChip1 for pathogenic variant screening and research across Qatari subpopulations, *n* = 2708 Qatari genomes were assessed by QChip1 for the number of variants of interest for SGD research and screening in the Qatari genetic subpopulations. After exclusion of common variants (minor allele frequency >0.05), variants in genes not containing ClinVar pathogenic variants, variants with a batch effect, and variants not observed in Qatar, *n* = 32,674variants of interest were analyzed. Population genetic analysis was conducted as described in Fig. 3. The Qatari individuals genotyped on QChip1 were stratified based on dominant ancestry cluster, without exclusion of admixed individuals. Shown is (left-to-right) each population with sample size, the median number of QChip1 variants per individual (homozygous, heterozygous, wild type, and missing) and median number of genes with one or more variants per individual.

^b^Populations include: Qatari (all Qatari) and subpopulations: QGP_PAR (Peninsular Arabs); QGP_GAR (General Arabs); QGP_WEP (Arabs of Wester Eurasia and Persia); QGP_SAS (South Asian Arabs); and QGP_AFR (African Arabs).

^c^Not included QGP_ADM, Admixed Arabs, see Table 3.

To help validate that QChip1 accurately detects known Qatari pathogenic variants, *n* = 140 variants identified as pathogenic either by the Hamad Medical Corporation (HMC) or by ClinVar were assessed in 2708 Qatari genomes by QChip1 (Table 5). There were *n* = 140 QChip1 pathogenic variants, including *n* = 140 (100%) present in ClinVar, *n* = 25 (18%) present in HMC, and *n* = 27 (19%) present in CAGS. Among these *n* = 140, *n* = 94 were only present in ClinVar, *n* = 19 were present in both HMC and ClinVar, *n* = 21 were present in ClinVar and CAGS but not HMC, and *n* = 6 present in all three pathogenic variant databases (ClinVar, HMC, CAGS). Among the *n* = 140 pathogenic variants, *n* = 3 were classified as “suspicious” based on high allele frequency (greater than 0.005)^27^. The three variants were previously reported in CAGS, HMC, or both, and appear to be truly pathogenic variants are enriched in the Qatari population due to founder effects, tribalism, consanguinity or a combination of these factors. One of these, NM_000071.2(CBS):c.1006C > T (p.Arg336Cys) linked to homocystinuria, is a well-documented founder variant in Qatar that was experimentally validated and is a priority for screening in the population^17,28^.

**Table 5:** Step 3: Known pathogenic variants of interest for Mendelian (single gene) disorder screening in Qatar using QChip1^a^.

Phenotype	DbSNP	Pathogenic DB^b^	Chr:Pos:Ref:Alt (GRCh38)	HGVS annotation assertion from ClinVar	Genotype Counts Hom:Het:WT	Qatar	Kuwait	GME	Iran	NYC	PR
Amyotrophic lateral sclerosis type 10	rs80356718	CV	1:11022209:A:G	NM_007375.3(TARDBP):c.800A > G (p.Asn267Ser)	0:6:2701	0.0011	0.0011	0.0012	0.0013	0.0000	0.0000
Homocystinuria due to methylene tetrahydrofolate reductase deficiency	rs776483190	CV	1:11802980:C:T	NM_005957.4(MTHFR):c.137G > A (p.Arg46Gln)	0:1:2705	0.0002	0.0000	0.0000	0.0000	0.0000	0.0000
Ehlers-Danlos syndrome, hydroxylysine-deficient	rs121913550	CV CAGS	1:11958627:C:T	NM_000302.4(PLOD1):c.955C > T (p.Arg319Ter)	0:4:2703	0.0007	0.0000	0.0000	0.0000	0.0000	0.0000
Glaucoma 1, open angle, A	rs74315339	CV	1:171652468:C:A	NM_000261.2(MYOC):c.144G > T (p.Gln48His)	0:6:2702	0.0011	0.0000	0.0005	0.0000	0.0000	0.0000
Central centrifugal cicatricial alopecia	rs142129409	CV	1:17262194:T:A	NM_016233.2(PADI3):c.335T > A (p.Leu112His)	0:2:2706	0.0004	0.0000	0.0045	0.0050	0.0000	0.0000
Central centrifugal cicatricial alopecia	rs139876092	CV	1:17267938:C:T	NM_016233.2(PADI3):c.628C > T (p.Arg210Trp)	0:2:2702	0.0004	0.0053	0.0010	0.0000	0.0000	0.0000
Central centrifugal cicatricial alopecia	rs139426141	CV	1:17270903:A:G	NM_016233.2(PADI3):c.856A > G (p.Thr286Ala)	0:18:2683	0.0033	0.0021	0.0025	0.0000	0.0133	0.0196
Central centrifugal cicatricial alopecia	rs144080386	CV	1:17270928:C:T	NM_016233.2(PADI3):c.881C > T (p.Ala294Val)	0:7:2697	0.0013	0.0021	0.0030	0.0050	0.0022	0.0000
Central centrifugal cicatricial alopecia	rs140482516	CV	1:17280704:C:T	NM_016233.2(PADI3):c.1669C> T (p.Arg557Trp)	0:3:2703	0.0006	0.0032	0.0015	0.0000	0.0066	0.0098
Central centrifugal cicatricial alopecia	rs34097903	CV	1:17280779:G:A	NM_016233.2(PADI3):c.1744G> A (p.Ala582Thr)	0:15:2692	0.0028	0.0011	0.0045	0.0006	0.0089	0.0000
Usher syndrome, type 2 A	rs777465132	CV	1:215758743:G:T	NM_206933.3(USH2A):c.11241C > A (p.Tyr3747Ter)	0:2:2704	0.0004	0.0000	0.0000	0.0000	0.0000	0.0000
Usher syndrome, type 2 A	rs746551311	CV	1:216196582:G:A	NM_206933.3(USH2A):c.4222C > T (p.Gln1408Ter)	0:2:2704	0.0004	0.0000	0.0000	0.0000	0.0000	0.0000
Porphyria cutanea tarda	rs121918066	CV	1:45015389:G:A	NM_000374.5(UROD):c.995G > A (p.Arg332His)	0:1:2702	0.0002	0.0000	0.0000	0.0013	0.0000	0.0000
Methylmalonic acidemia with homocystinuria	rs796051995	CV	1:45507491:C:T	NM_015506.3(MMACHC):c.217C > T (p.Arg73Ter)	0:1:2704	0.0002	0.0000	0.0000	0.0000	0.0000	0.0000
Leber congenital amaurosis 2	rs61752871	CV	1:68444858:G:A	NM_000329.3(RPE65):c.271C > T (p.Arg91Trp)	0:2:2706	0.0004	0.0000	0.0000	0.0006	0.0000	0.0000
Advanced sleep phase syndrome, familial, 3	rs139315125	CV	1:7809900:A:G	NM_016831.3(PER3):c.1247A > G (p.His416Arg)	0:7:2701	0.0013	0.0021	0.0005	0.0038	0.0000	0.0000
Parkinson disease 7	rs74315352	CV	1:7984930:A:C	NM_007262.5(PARK7):c.446A > C (p.Asp149Ala)	0:1:2704	0.0002	0.0000	0.0000	0.0000	0.0000	0.0000
Stargardt disease*	rs1800553	CV CAGS	1:94008251:C:T	NM_000350.2(ABCA4):c.[5512C > G;5882 G > A]	3:106:2594	0.0207	0.0170	0.0211	0.0256	0.0089	0.0098
Stargardt disease	rs61750155	CV CAGS	1:94021695:G:T	NM_000350.3(ABCA4):c.4793C > A (p.Ala1598Asp)	0:3:2698	0.0006	0.0000	0.0010	0.0006	0.0000	0.0000
Leber congenital amaurosis 9	rs150726175	CV	1:9982630:G:A	NM_022787.4(NMNAT1):c.769G > A (p.Glu257Lys)	0:1:2705	0.0002	0.0000	0.0015	0.0013	0.0000	0.0000
Glycogen storage disease type III	rs775685508	CV	1:99916603:G:T	NM_000642.3(AGL):c.4353G > T (p.Trp1451Cys)	0:4:2703	0.0007	0.0000	0.0000	0.0000	0.0000	0.0000
Nemaline myopathy 2	rs886041851	CV HMC	2:151610867:C:T	NM_001271208.2(NEB):c.11806-1G > A	0:16:2691	0.0030	0.0000	0.0000	0.0000	0.0000	0.0000
Hyperphosphatemic familial tumoral calcinosis 1	rs137853086	CV	2:165770217:G:A	NM_004482.4(GALNT3):c.484C > T (p.Arg162Ter)	0:1:2707	0.0002	0.0000	0.0005	0.0000	0.0000	0.0000
Biotin-thiamine-responsive basal ganglia disease	rs121917884	CV HMC	2:227688216:T:C	NM_025243.4(SLC19A3):c.1264A > G (p.Thr422Ala)	0:6:2698	0.0011	0.0000	0.0000	0.0000	0.0000	0.0000
Deafness, autosomal recessive 9	rs397515591	CV HMC	2:26477725:C:A	NM_194248.3(OTOF):c.2239G > T (p.Glu747Ter)	0:5:2703	0.0009	0.0000	0.0000	0.0000	0.0000	0.0000
3-Oxo-5 alpha-steroid delta 4-dehydrogenase deficiency	rs9332967	CV	2:31526224:C:T	NM_000348.4(SRD5A2):c.737G > A (p.Arg246Gln)	0:1:2705	0.0002	0.0000	0.0000	0.0000	0.0000	0.0000
3-Oxo-5 alpha-steroid delta 4-dehydrogenase deficiency	rs763296857	CV	2:31529427:T:C	NM_000348.4(SRD5A2):c.578A > G (p.Asn193Ser)	0:1:2704	0.0002	0.0000	0.0000	0.0000	0.0000	0.0000
Glaucoma 3, primary congenital, A	rs28936700	CV CAGS	2:38075207:C:T	NM_000104.3(CYP1B1):c.182G > A (p.Gly61Glu)	0:1:2705	0.0002	0.0000	0.0031	0.0044	0.0000	0.0098
Sitosterolemia	rs137852988	CV	2:43875377:G:A	NM_022437.3(ABCG8):c.1720G> A (p.Gly574Arg)	0:5:2699	0.0009	0.0042	0.0000	0.0000	0.0000	0.0000
Achromatopsia	rs141386891	CV	2:98396449:C:T	NM_001298.3(CNGA3):c.1279C > T (p.Arg427Cys)	0:3:2704	0.0006	0.0000	0.0000	0.0000	0.0000	0.0000
Fanconi anemia, complementation group D2	rs112832879	CV	3:10043483:G:A	NM_033084.5(FANCD2):c.990-1G > A	0:1:2706	0.0002	0.0000	0.0000	0.0000	0.0000	0.0000
Macular dystrophy, vitelliform, 5	rs199867882	CV CAGS	3:101231117:G:A	NM_016247.4(IMPG2):c.3262C > T (p.Arg1088Ter)	0:2:2705	0.0004	0.0011	0.0000	0.0000	0.0000	0.0000
Retinitis pigmentosa 61	rs775098953	CV	3:150928174:A:C	NM_174878.3(CLRN1):c.461T > G (p.Leu154Trp)	0:1:2705	0.0002	0.0000	0.0000	0.0000	0.0000	0.0000
Biotinidase deficiency	rs397514369	CV	3:15644413:G:A	NM_001370658.1(BTD):c.497G > A (p.Cys166Tyr)	0:5:2702	0.0009	0.0011	0.0000	0.0000	0.0000	0.0000
Biotinidase deficiency	rs13078881	CV	3:15645186:G:C	NM_000060.2(BTD):c.[470G > A;1330 G > C]	4:135:2557	0.0265	0.0000	0.0272	0.0294	0.0360	0.0392
Biotinidase deficiency	rs138818907	CV CAGS	3:15645345:C:T	NM_001370658.1(BTD):c.1429C > T (p.Pro477Ser)	0:5:2701	0.0009	0.0000	0.0010	0.0000	0.0000	0.0000
Brugada syndrome	rs199473101	CV	3:38606682:C:T	NM_198056.2(SCN5A):c.1127G > A (p.Arg376His)	0:1:2707	0.0002	0.0000	0.0000	0.0000	0.0000	0.0000
Chanarin-Dorfman Syndrome	rs104893676	CV	3:43691011:G:A	NM_016006.6(ABHD5):c.19G > A (p.Glu7Lys)	0:2:2704	0.0004	0.0000	0.0000	0.0000	0.0000	0.0000
Dystrophic epidermolysis bullosa	rs756217590	CV HMC	3:48583161:C:T	NM_000094.3(COL7A1):c.4448G > A (p.Gly1483Asp)	0:1:2706	0.0002	0.0000	0.0000	0.0000	0.0000	0.0000
Spastic paraplegia 56, autosomal recessive	rs397514513	CV HMC	4:107945426:A:T	NM_183075.3(CYP2U1):c.947A > T (p.Asp316Val)	0:1:2702	0.0002	0.0032	0.0000	0.0000	0.0000	0.0000
Hypofibrinogenemia	rs121909607	CV	4:154589513:C:T	NM_021871.4(FGA):c.104G > A (p.Arg35His)	0:3:2703	0.0006	0.0000	0.0000	0.0000	0.0000	0.0000
Bietti crystalline corneoretinal dystrophy	rs199476187	CV	4:186194568:G:A	NM_207352.4(CYP4V2):c.283G > A (p.Gly95Arg)	0:2:2704	0.0004	0.0000	0.0000	0.0000	0.0000	0.0000
Hereditary factor XI deficiency disease	rs121965063	CV CAGS	4:186274193:G:T	NM_000128.3(F11):c.403G > T (p.Glu135Ter)	0:7:2700	0.0013	0.0011	0.0010	0.0006	0.0000	0.0000
Hereditary factor XI deficiency disease	rs542967227	CV	4:186285765:G:A	NM_000128.3(F11):c.1432G > A (p.Gly478Arg)	0:1:2706	0.0002	0.0000	0.0000	0.0000	0.0000	0.0000
Hypogonadotropic hypogonadism 7 with or without anosmia	rs104893836	CV	4:67754019:T:C	NM_000406.3(GNRHR):c.317A > G (p.Gln106Arg)	0:4:2703	0.0007	0.0011	0.0055	0.0019	0.0089	0.0000
Hyaline fibromatosis syndrome	rs886041401	CV CAGS	4:80072427:A:G	NM_058172.6(ANTXR2):c.134T > C (p.Leu45Pro)	0:4:2701	0.0007	0.0000	0.0000	0.0000	0.0000	0.0000
Hartnup disorder	rs121434347	CV	5:1213517:C:T	NM_001003841.3(SLC6A19):c.718 C > T (p.Arg240Ter)	0:4:2703	0.0007	0.0011	0.0005	0.0000	0.0000	0.0000
Renal carnitine transport defect	rs72552724	CV HMC	5:132370055:G:T	NM_003060.4(SLC22A5):c.83G > T (p.Ser28Ile)	0:5:2701	0.0009	0.0032	0.0000	0.0000	0.0000	0.0000
Primary systemic carnitine deficiency	rs886041277	CV HMC	5:132378379:G:A	NM_003060.4(SLC22A5):c.395G > A (p.Trp132Ter)	0:1:2706	0.0002	0.0000	0.0000	0.0000	0.0000	0.0000
Nijmegen breakage syndrome-like disorder	rs772468452	CV	5:132595719:C:T	NM_005732.4(RAD50):c.2116C > T (p.Arg706Ter)	0:1:2707	0.0002	0.0000	0.0000	0.0000	0.0000	0.0000
Primary ciliary dyskinesia	rs761622153	CV HMC	5:13841112:G:A	NM_001277115.2(DNAH11):c.5924 + 1 G > C	0:71:2634	0.0131	0.0110	0.0000	0.0019	0.0000	0.0000
Seizures, cortical blindness, and microcephaly syndrome	rs863225243	CV HMC	5:141528456:G:A	NM_005219.5(DIAPH1):c.3145C > T (p.Arg1049Ter)	0:2:2704	0.0004	0.0000	0.0000	0.0000	0.0000	0.0000
Ehlers-Danlos syndrome, spondylodysplastic type, 1	rs28937869	CV	5:177608994:C:T	NM_007255.3(B4GALT7):c.808C > T (p.Arg270Cys)	0:1:2703	0.0002	0.0000	0.0000	0.0000	0.0000	0.0000
Mucopolysaccharidosis type 6	rs771296632	CV	5:78839361:G:A	NM_000046.5(ARSB):c.1208C > G (p.Ser403Ter)	0:1:2705	0.0002	0.0000	0.0000	0.0000	0.0000	0.0000
Hypophosphatemic rickets, autosomal recessive, 2	rs373044722	CV	6:131872926:C:T	NM_006208.3(ENPP1):c.1441C > T (p.Arg481Trp)	0:1:2705	0.0002	0.0000	0.0000	0.0000	0.0000	0.0000
non-classical congenital adrenal hyperplasia	rs776989258	CV	6:32041093:C:T	NM_000500.9(CYP21A2):c.1447C > T (p.Pro483Ser)	0:4:2701	0.0007	0.0000	0.0000	0.0057	0.0000	0.0000
Leukodystrophy, hypomyelinating, 11	rs141156009	CV	6:43520961:C:T	NM_203290.4(POLR1C):c.835C > T (p.Arg279Trp)	0:4:2704	0.0007	0.0000	0.0000	0.0000	0.0000	0.0000
Autosomal recessive polycystic kidney disease	rs794727566	CV	6:52024750:A:G	NM_138694.4(PKHD1):c.5060T > C (p.Ile1687Thr)	0:2:2702	0.0004	0.0000	0.0000	0.0000	0.0000	0.0000
Autosomal recessive polycystic kidney disease	rs773136605	CV	6:52043102:C:T	NM_138694.4(PKHD1):c.2854G > A (p.Gly952Arg)	0:3:2704	0.0006	0.0000	0.0000	0.0000	0.0000	0.0000
Autosomal recessive polycystic kidney disease	rs398124478	CV	6:52048558:G:A	NM_138694.4(PKHD1):c.	0:1:2705	0.0002	0.0000	0.0000	0.0000	0.0000	0.0000
Retinitis pigmentosa	rs930421180	CV	6:64591822:G:A	NM_001142800.2(EYS):c.4045C > T (p.Arg1349Ter)	0:1:2703	0.0002	0.0000	0.0000	0.0000	0.0000	0.0000
Pendred syndrome	rs111033348	CV	7:107674326:C:T	NM_000441.1(SLC26A4):c.578C > T	0:3:2704	0.0006	0.0000	0.0000	0.0000	0.0000	0.0000
Pendred syndrome	rs111033256	CV CAGS	7:107675060:T:A	NM_000441.2(SLC26A4):c.716T > A (p.Val239Asp)	0:7:2699	0.0013	0.0000	0.0010	0.0000	0.0000	0.0000
Congenital secretory diarrhea, chloride type	rs121913032	CV	7:107791059:C:A	NM_000111.2(SLC26A3):c.559G > T (p.Gly187Ter)	0:6:2701	0.0011	0.0053	0.0000	0.0006	0.0022	0.0000
Maple syrup urine disease, type 3	rs121964990	CV HMC CAGS	7:107915506:G:T	NM_000108.5(DLD):c.685G > T (p.Gly229Cys)	0:14:2689	0.0026	0.0000	0.0010	0.0000	0.0022	0.0000
Cystic fibrosis	rs121909005	CV CAGS	7:117587801:T:G	NM_000492.4(CFTR):c.1647T> G (p.Ser549Arg)	0:1:2706	0.0002	0.0000	0.0005	0.0006	0.0000	0.0000
Cystic fibrosis	rs75096551	CV CAGS	7:117606754:G:A	NM_000492.3(CFTR):c.2988 + 1 G > A	0:2:2701	0.0004	0.0000	0.0000	0.0006	0.0000	0.0000
Cystic fibrosis	rs121909043	CV	7:117667029:C:G	NM_000492.3(CFTR):c.4364C > G (p.Ser1455Ter)	0:1:2705	0.0002	0.0000	0.0000	0.0000	0.0000	0.0000
Primary ciliary dyskinesia	rs886039340	CV HMC	7:21687528:G:C	NM_001277115.2(DNAH11):c.5924 + 1 G > C	0:15:2693	0.0028	0.0000	0.0000	0.0000	0.0000	0.0000
Deficiency of aromatic-L-amino-acid decarboxylase	rs201951824	CV	7:50476625:C:T	NM_001082971.2(DDC):c.1040G > A (p.Arg347Gln)	0:2:2702	0.0004	0.0000	0.0010	0.0000	0.0000	0.0000
Argininosuccinate lyase deficiency	rs367543005	CV CAGS	7:66089693:C:T	NM_000048.4(ASL):c.1060C > T (p.Gln354Ter)	0:1:2707	0.0002	0.0000	0.0000	0.0000	0.0000	0.0000
Cohen syndrome	rs140353201	CV	8:99467566:C:T	NM_017890.4(VPS13B):c.3598C > T (p.Arg1200Ter)	0:1:2706	0.0002	0.0000	0.0000	0.0000	0.0000	0.0000
Rare genetic deafness	rs779760634	CV	9:114423524:C:T	NM_015404.4(WHRN):c.1417-1G > A	0:1:2701	0.0002	0.0000	0.0000	0.0000	0.0000	0.0000
Walker-Warburg congenital muscular dystrophy	rs776061161	CV	9:131510063:G:A	NM_001077365.2(POMT1):c.699 + 67 G > A	0:2:2704	0.0004	0.0000	0.0000	0.0000	0.0000	0.0000
Deficiency of UDPglucose-hexose-1-phosphate uridylyltransferase	rs111033735	CV	9:34648371:G:A	NM_000155.4(GALT):c.602G > A (p.Arg201His)	0:1:2706	0.0002	0.0000	0.0000	0.0000	0.0000	0.0000
Primary hyperoxaluria, type II	rs180177314	CV	9:37429732:G:A	NM_012203.2(GRHPR):c.494G > A (p.Gly165Asp)	0:3:2703	0.0006	0.0000	0.0000	0.0006	0.0000	0.0000
Pontocerebellar hypoplasia, type 1b	rs387907196	CV	9:37784953:C:G	NM_016042.4(EXOSC3):c.92G > C (p.Gly31Ala)	0:1:2703	0.0002	0.0000	0.0000	0.0000	0.0000	0.0000
Testosterone 17-beta-dehydrogenase deficiency	rs119481077	CV CAGS	9:96254907:G:A	NM_000197.2(HSD17B3):c.238C > T (p.Arg80Trp)	0:2:2703	0.0004	0.0000	0.0015	0.0000	0.0000	0.0000
Histiocytosis-lymphadenopathy plus syndrome	rs397515429	CV	10:71362337:G:A	NM_018344.6(SLC29A3):c.1157G > A (p.Arg386Gln)	0:1:2702	0.0002	0.0000	0.0000	0.0000	0.0000	0.0000
Glucose-6-phosphate transport defect	rs121908979	CV	11:119024957:G:A	NM_001164278.2(SLC37A4):c.1309 C > T (p.Arg437Ter)	0:1:2707	0.0002	0.0000	0.0000	0.0000	0.0000	0.0000
Beta-thalassemia	rs34716011	CV HMC	11:5226974:C:T	NM_000518.5(HBB):c.48G > A (p.Trp16Ter)	0:1:2705	0.0002	0.0000	0.0000	0.0000	0.0000	0.0000
Joubert syndrome 16	rs387907133	CV CAGS	11:61368600:C:T	NM_016464.5(TMEM138):c.380C > T (p.Ala127Val)	0:2:2699	0.0004	0.0000	0.0000	0.0000	0.0000	0.0000
Niemann-Pick disease, type B	rs120074126	CV HMC	11:6393620:C:T	NM_000543.5(SMPD1):c.1267C > T (p.His423Tyr)	0:1:2705	0.0002	0.0000	0.0000	0.0000	0.0000	0.0000
Mucolipidosis	rs34940801	CV	12:101757571:C:T	NM_024312.5(GNPTAB):c.3335+ 1 G > A	0:4:2702	0.0007	0.0000	0.0000	0.0000	0.0022	0.0000
Phenylketonuria	rs5030857	CV CAGS	12:102840507:G:A	NM_000277.3(PAH):c.1208C > T (p.Ala403Val)	0:2:2702	0.0004	0.0000	0.0010	0.0000	0.0022	0.0000
Phenylketonuria	rs5030853	CV	12:102851701:C:A	NM_000277.3(PAH):c.898G > T (p.Ala300Ser)	0:7:2698	0.0013	0.0000	0.0015	0.0025	0.0000	0.0000
Vitamin B12-responsive methylmalonic acidemia type cblB	rs763935916	CV CAGS	12:109568864:C:A	NM_052845.4(MMAB):c.197-1G > T	0:6:2700	0.0011	0.0000	0.0000	0.0006	0.0000	0.0000
Glycogen storage disease due to hepatic glycogen synthase deficiency	rs121918419	CV	12:21568952:G:A	NM_021957.4(GYS2):c.736C > T (p.Arg246Ter)	0:1:2705	0.0002	0.0011	0.0015	0.0006	0.0000	0.0000
Glycogen storage disease due to hepatic glycogen synthase deficiency	rs201157731	CV	12:21574275:G:A	NM_021957.4(GYS2):c.547C > T (p.Gln183Ter)	0:4:2703	0.0007	0.0000	0.0000	0.0000	0.0000	0.0000
Myopathy, lactic acidosis, and sideroblastic anemia 2	rs587777214	CV	12:32750744:G:A	NM_001040436.3(YARS2):c.1078 C > T (p.Arg360Ter)	0:1:2707	0.0002	0.0000	0.0000	0.0000	0.0000	0.0000
Parkinson disease 8, autosomal dominant	rs34637584	CV	12:40340400:G:A	NM_198578.4(LRRK2):c.6055G > A (p.Gly2019Ser)	0:4:2704	0.0007	0.0021	0.0050	0.0000	0.0000	0.0000
Bailey-Bloch congenital myopathy	rs140291094	CV	12:57244322:C:G	NM_145064.3(STAC3):c.851G > C (p.Trp284Ser)	0:1:2703	0.0002	0.0000	0.0000	0.0000	0.0000	0.0000
von Willebrand disease, type 2a	rs41276738	CV	12:6034812:C:T	NM_000552.4(VWF):c.2561G > A (p.Arg854Gln)	0:3:2704	0.0006	0.0000	0.0015	0.0006	0.0022	0.0098
Temtamy syndrome	rs587776954	CV HMC	12:6944122:A:G	NM_138425.4(C12orf57):c.1A > G (p.Met1Val)	0:6:2700	0.0011	0.0074	0.0000	0.0000	0.0000	0.0000
Peroxisome biogenesis disorder 2A (Zellweger)	rs61752138	CV	12:7209700:T:G	NM_001131025.1(PEX5):c.1578T > G (p.Asn526Lys)	0:2:2704	0.0004	0.0000	0.0000	0.0000	0.0000	0.0000
Factor VII deficiency	rs121964926	CV	13:113118698:G:A	NM_019616.4(F7):c.1025G > A (p.Arg342Gln)	0:1:2707	0.0002	0.0000	0.0005	0.0000	0.0000	0.0000
Deafness, autosomal recessive 1A	rs774518779	CV HMC CAGS	13:20189076:C:T	NM_004004.6(GJB2):c.506G > A (p.Cys169Tyr)	0:4:2702	0.0007	0.0000	0.0000	0.0000	0.0000	0.0000
Deafness, autosomal recessive 1A	rs104894396	CV	13:20189511:C:T	NM_004004.6(GJB2):c.71G > A (p.Trp24Ter)	0:5:2702	0.0009	0.0000	0.0000	0.0000	0.0000	0.0000
Deafness, autosomal recessive 1A	rs80338940	CV CAGS	13:20192782:C:T	NM_004004.6(GJB2):c.-23 + 1 G > A	0:2:2705	0.0004	0.0000	0.0000	0.0000	0.0000	0.0000
Deafness, autosomal dominant 3b	rs104894414	CV	13:20223467:G:A	NM_001110219.3(GJB6):c.14C > T (p.Thr5Met)	0:6:2699	0.0011	0.0000	0.0000	0.0000	0.0000	0.0000
Aicardi Goutieres syndrome 2	rs75184679	CV HMC CAGS	13:50945445:G:A	NM_024570.4(RNASEH2B):c.529G > A (p.Ala177Thr)	0:3:2704	0.0006	0.0011	0.0015	0.0006	0.0000	0.0000
Leber congenital amaurosis 6	rs554396590	CV	14:21303542:C:T	NM_020366.3(RPGRIP1):c.799C > T (p.Arg267Ter)	0:1:2703	0.0002	0.0000	0.0000	0.0000	0.0000	0.0000
Galactosylceramide beta-galactosidase deficiency	rs199847983	CV HMC	14:87968386:C:T	NM_000153.4(GALC):c.857G > A (p.Gly286Asp)	0:14:2692	0.0026	0.0011	0.0000	0.0013	0.0000	0.0000
Alpha-1-antitrypsin deficiency	rs28931569	CV	14:94383044:A:G	NM_001127701.1(SERPINA1):c.194 T > C (p.Leu65Pro)	0:2:2702	0.0004	0.0000	0.0010	0.0000	0.0000	0.0000
Mosaic variegated aneuploidy syndrome 1	rs28989186	CV	15:40176672:C:T	NM_001211.5(BUB1B):c.580C > T (p.Arg194Ter)	0:3:2704	0.0006	0.0000	0.0000	0.0000	0.0000	0.0000
Limb-girdle muscular dystrophy, type 2A	rs147764579	CV	15:42401752:G:A	NM_000070.3(CAPN3):c.1466G > A (p.Arg489Gln)	0:1:2706	0.0002	0.0021	0.0015	0.0000	0.0044	0.0000
Peeling skin syndrome 2	rs112292549	CV	15:43260151:C:A	NM_201631.4(TGM5):c.337G > T (p.Gly113Cys)	0:3:2704	0.0006	0.0011	0.0000	0.0000	0.0044	0.0098
Tay-Sachs disease	rs786204721	CV CAGS	15:72375971:A:G	NM_000520.6(HEXA):c.2T > C (p.Met1Thr)	0:2:2702	0.0004	0.0000	0.0000	0.0000	0.0000	0.0000
Amyotrophic lateral sclerosis type 6	rs387906628	CV	16:31185031:G:A	NM_004960.3(FUS):c.616G > A (p.Gly206Ser)	0:2:2701	0.0004	0.0000	0.0000	0.0006	0.0000	0.0000
Meier-Gorlin syndrome 3	rs146795505	CV	16:46689707:T:C	NM_014321.4(ORC6):c.2T > C (p.Met1Thr)	0:1:2705	0.0002	0.0000	0.0000	0.0000	0.0000	0.0098
Polymicrogyria, bilateral frontoparietal	rs121908462	CV	16:57651247:C:T	NM_201525.4(ADGRG1):c.112C > T (p.Arg38Trp)	0:2:2706	0.0004	0.0000	0.0000	0.0000	0.0022	0.0000
Polymicrogyria, bilateral frontoparietal	rs121908465	CV	16:57651407:G:C	NM_201525.4(ADGRG1):c.272G > C (p.Cys91Ser)	0:1:2707	0.0002	0.0000	0.0000	0.0000	0.0000	0.0000
Spermatogenic failure 31	rs140352254	CV	16:72122957:G:A	NM_031293.3(PMFBP1):c.2725C > T (p.Arg909Ter)	0:2:2704	0.0004	0.0000	0.0000	0.0000	0.0000	0.0000
Congenital disorder of glycosylation	rs28936415	CV CAGS	16:8811153:G:A	NM_000303.3(PMM2):c.422G > A (p.Arg141His)	0:8:2688	0.0015	0.0000	0.0046	0.0019	0.0000	0.0000
Fanconi anemia, complementation group A	rs769479800	CV	16:89816614:A:G	NM_000135.4(FANCA):c.2T > C (p.Met1Thr)	0:1:2702	0.0002	0.0000	0.0000	0.0000	0.0000	0.0000
Neurofibromatosis, type 1	rs137854562	CV HMC	17:31235623:C:T	NM_000267.3(NF1):c.3721C > T (p.Arg1241Ter)	0:1:2707	0.0002	0.0000	0.0000	0.0000	0.0000	0.0000
Canavan Disease, Familial Form	rs766328537	CV	17:3476238:G:A	NM_000049.3(ASPA):c.79G > A (p.Gly27Arg)	0:3:2704	0.0006	0.0000	0.0000	0.0000	0.0000	0.0000
Mucopolysaccharidosis, MPS-III-B	rs104894595	CV	17:42543568:C:T	NM_000263.4(NAGLU):c.1562C > T (p.Pro521Leu)	0:1:2700	0.0002	0.0000	0.0000	0.0000	0.0000	0.0000
Spherocytosis type 4, due to band 3, Cape Town	rs28929480	CV	17:44260716:C:T	NM_000342.3(SLC4A1):c.268G > A (p.Glu90Lys)	0:1:2702	0.0002	0.0011	0.0000	0.0000	0.0000	0.0000
Pyridoxal phosphate-responsive seizures	rs773450573	CV HMC	17:47946682:G:A	NM_018129.4(PNPO):c.686G > A (p.Arg229Gln)	0:1:2705	0.0002	0.0000	0.0000	0.0000	0.0000	0.0000
Sarcoglycanopathy	rs371675217	CV HMC	17:50167431:G:A	NM_000023.4(SGCA):c.101G > A (p.Arg34His)	0:2:2704	0.0004	0.0000	0.0000	0.0000	0.0000	0.0000
Sarcoglycanopathy	rs143570936	CV	17:50169246:G:A	NM_000023.4(SGCA):c.739G > A (p.Val247Met)	0:4:2704	0.0007	0.0000	0.0000	0.0006	0.0000	0.0000
Meckel-Gruber syndrome	rs786205508	CV HMC CAGS	17:58208542:G:A	NM_001165927.1(MKS1):c.1036 C > T (p.Gln346Ter)	0:1:2704	0.0002	0.0000	0.0000	0.0000	0.0000	0.0000
Glycogen storage disease, type II	rs778418246	CV	17:80113002:G:A	NM_000152.5(GAA):c.2015G> A (p.Arg672Gln)	0:1:2705	0.0002	0.0000	0.0000	0.0000	0.0000	0.0000
Niemann-Pick disease type C1	rs759826138	CV	18:23539394:G:A	NM_000271.5(NPC1):c.2872C > T (p.Arg958Ter)	0:4:2703	0.0007	0.0000	0.0000	0.0000	0.0000	0.0000
Junctional epidermolysis bullosa	rs886039412	CV HMC	18:23928766:G:A	NM_000227.5(LAMA3):c.3609 + 1 G > A	0:1:2705	0.0002	0.0000	0.0000	0.0000	0.0000	0.0000
Amyloid Cardiomyopathy, Transthyretin-related	rs76992529	CV	18:31598655:G:A	NM_000371.4(TTR):c.424G > A (p.Val142Ile)	0:8:2695	0.0015	0.0000	0.0005	0.0000	0.0022	0.0000
Vici syndrome	rs767638289	CV	18:45954507:G:A	NM_020964.3(EPG5):c.895C > T (p.Arg299Ter)	0:2:2703	0.0004	0.0000	0.0000	0.0000	0.0000	0.0000
Obesity, autosomal dominant	rs121913560	CV	18:60371842:T:C	NM_005912.3(MC4R):c.508 A > G (p.Ile170Val)	0:14:2693	0.0026	0.0011	0.0000	0.0000	0.0000	0.0000
Familial hypercholesterolemia 1	rs148698650	CV	19:11107403:G:A	NM_000527.4(LDLR):c.829G > T (p.Glu277Ter)	0:2:2704	0.0004	0.0011	0.0010	0.0031	0.0000	0.0000
Mental retardation, autosomal recessive 3	rs876657679	CV	19:13913639:G:T	NM_017721.5(CC2D1A):c.748 + 1 G > T	0:3:2702	0.0006	0.0000	0.0000	0.0000	0.0000	0.0000
Autosomal recessive congenital ichthyosis 5	rs118203937	CV HMC CAGS	19:15540506:G:A	NM_173483.4(CYP4F22):c.728G > A (p.Arg243His)	2:1:2697	0.0009	0.0000	0.0000	0.0000	0.0000	0.0000
Autosomal recessive congenital ichthyosis 5	rs118203935	CV CAGS	19:15549170:C:T	NM_173483.4(CYP4F22):c.1303C > T (p.His435Tyr)	0:1:2706	0.0002	0.0000	0.0005	0.0000	0.0000	0.0000
Nephrotic syndrome, type 9	rs398122978	CV CAGS	19:40705140:G:A	NM_024876.4(COQ8B):c.532C > T (p.Arg178Trp)	0:1:2697	0.0002	0.0000	0.0000	0.0000	0.0000	0.0000
Graves disease	rs775644973	CV	20:1002085:C:T	NM_001029871.4(RSPO4):c.79 + 1 G > A	0:3:2703	0.0006	0.0000	0.0000	0.0006	0.0000	0.0000
Homocystinuria	rs121964972	CV	21:43060528:G:A	NM_000071.2(CBS):c.1058C > T (p.Thr353Met)	0:1:2704	0.0002	0.0000	0.0000	0.0000	0.0000	0.0000
Homocystinuria*	rs398123151	CV HMC CAGS	21:43062344:G:A	NM_000071.2(CBS):c.1006C > T (p.Arg336Cys)	1:31:2674	0.0061	0.0000	0.0000	0.0000	0.0000	0.0000
microcephalic osteodysplastic primordial dwarfism type 2	rs777830265	CV HMC	21:46355533:C:T	NM_006031.6(PCNT):c.1843C > T (p.Gln615Ter)	0:1:2706	0.0002	0.0000	0.0000	0.0000	0.0000	0.0000
Deficiency of beta-ureidopropionase	rs747539101	CV	22:24520469:G:A	NM_016327.3(UPB1):c.873 + 1 G > A	0:8:2695	0.0015	0.0000	0.0000	0.0013	0.0000	0.0000
Acute infantile liver failure due to synthesis defect of mtDNA-encoded proteins	rs387907022	CV	22:46353829:G:A	NM_018006.5(TRMU):c.835G > A (p.Val279Met)	0:1:2705	0.0002	0.0000	0.0000	0.0000	0.0000	0.0000

^a^As examples of the use of QChip1, the 2,708 Qatari genomes were assessed for 140 pathogenic variants known to be present in the Qatari genome from the Hamad Medical Corporation genetic screening database. All of these variants were predicted by ClinVar to be pathogenic and by and SnpEff to have moderate or high impact on protein function. From left-to-right is the disease name, DbSNP rsID, list of databases where variant is found, genomic coordinates (GRCh38 reference), and the alternate allele frequency in Qatar, Kuwait, NYC, and Puerto Rico. From this analysis the Qatar, USA and Puerto Rico genetic subgroups are combined for each location. Table 5 is a subset of the *n* = 32,674 variants genotyped based on computationally predicted value for SGD research or screening; the complete dataset is in Supplementary Table 3. Variants at unusually elevated allele frequency in Qatar have an “*” next to the disease name.

^b^Each variant rsID was queried in three databases, including the ClinVar website (CV) (accessed May 2021), the Center for Arab Genetics Disorders (CAGS), and the Hamad Medical Corporation database of SGD disease case reports. For each record, the disease name and HGVS annotation were taken from ClinVar, and variants that were not described as “pathogenic” in ClinVar or did not have a phenotype described were excluded.

A major question for the future of QChip is the applicability of the variant list in other GME populations. In order to begin to answer this question, the QChip1 variant list was looked up in four datasets, including sequencing data from CAGS, Kuwait, Iran, and a collection across the GME (GME Variome)^29–32^. Out of the *n* = 140 pathogenic variants in Qatar genotyped by QChip1, 50%% (*n* = 70) were observed in one or more of the 4 GME datasets, including *n* = 28 (20%) in Kuwait, *n* = 32 (23%) in Iran, and *n* = 37 (26%) in the GME Variome. As expected, only *n* = 8 (6%) were observed in Puerto Rico and *n* = 16 (13%) were observed in NYC (Table 6). Based on these data, the utility of QChip1 was higher in GME than in the Americas; however, half the variants were unique to Qatar, and thus each GME nation (such as Kuwait and Iran) could benefit from a custom design.

**Table 6:** QChip1 pathogenic variants in genomics knowledgebases^a^.

Knowledgebase	Sample size for allele frequency	QChip1 pathogenic variants	
		*n*	%
QChip1	2708	140	100
ClinVar		140	100
Hamad Medical Corporation (HMC)		25	18
Center for Arab Genetic Studies (CAGS)		27	19
Dasman Diabetes Institute (DDI, Kuwait)	540	28	20
GME Variome	886	37	26
Iranome (Iran)	800	32	23
New York City (NYC)	226	16	11
Puerto Ricans (PR)	51	8	6
Anywhere		87	62
Middle East (CAGS, Kuwait, GME, Iran)		70	50

^a^In order to quantify the utility of QChip1 for single gene (Mendelian) disorder screening outside of Qatar, the presence and (when available) allele frequency of each variant in Table 5 was checked in seven datasets, including three produced by this research team (HMC, NYC, PR) and four externally obtained [CAGS (http://cags.org.ae/), Dasman Diabetes Institute, GME Variome (http://igm.ucsd.edu/gme/data-browser.php), Iranome (http://www.iranome.ir/)]. Only the DDI, GME, and Iranome datasets had allele frequency data. Shown is the name of the knowledgebase, the sample size when available, and the QChip1 pathogenic variants found in the knowledgebase, including number and percentage of 140 total on QChip1 (Table 5).

^2^For datasets where allele frequency is available, the variant is counted as “present” if the frequency was great than zero. For datasets where allele frequency is not available, the variant is counted as “present” if a query of the dataset found the variant. The bottom two rows show aggregate data, where the “anywhere” row indicates variants present in any of the seven datasets (HMC, CAGS, Kuwait, GME, Iran, NYC, PR), and the “Middle East” row indicates variants present in the Middle Eastern datasets (CAGS, DDI, GME, Iran).

All 140 of the pathogenic variants were accurately detected by QChip1 and were described in Table 5; for additional variants of interest for SGD research on QChip1 assessed on 2,708 Qatari genomes, see Supplementary Table 3. In Table 5 pathogenic variants were identified in CBS, a gene linked to homocystinuria (rs398123151 and rs121964972, 1 homozygote and 32 heterozygotes combined, 0.62% genomes), nemaline myopathy (rs886041851,16 heterozygotes, 0.3% genomes), and factor XI deficiency (rs121965063, 0.13% genomes). Relevant to these observations, all 2708 genomes tested were from the general medical clinic and general population, not from referrals to genetic disease clinics, and hence these data were interpreted as representative of the general population of Qatar.

Examination of the distribution of types of functional variants identified by QChip1 in the Qatari genome, the majority of variants of interest for research that were computationally predicted to have “high impact” were involved in structural interaction, which currently would be considered “benign” or “uncertain significance” by ACMG standards and ClinVar. The most common class of variants of interest for research that were computationally predicted “moderate” impact were missense variants (Supplementary Table 4). In some cases, the SnpEff annotation was different from the ClinVar annotation for a pathogenic variant, typically in situations where multiple transcripts lead to multiple alternative annotations for a varant and SnpEff is not aware of the “canonical” annotation in the literature, such as for NM_000071.2(CBS):c.1006C > T (p.Arg336Cys), which SnpEff correctly annotated on the transcript as c.1006C > T but did not provide the amino-acid change, but rather annotated it as “structural_interaction_variant”.

**Fig. 1:**
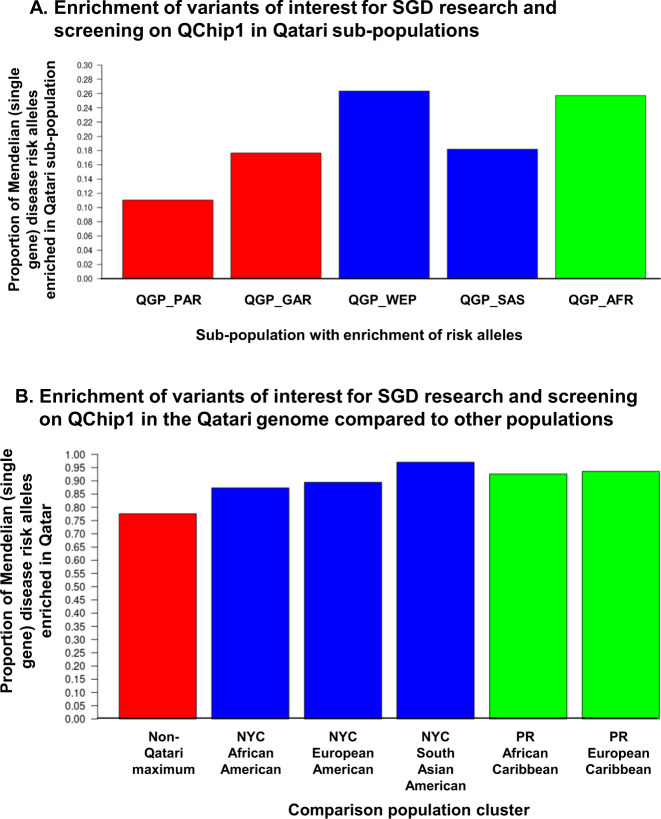
Population distribution of QChip1 variants observed in Qatar. In order to demonstrate the population-specific value of QChip1, the risk alleles that were discovered by genome/exome sequencing, prioritized in the knowledgebase, included in the array design, successfully genotyped, and observed in array data for at least one of *n* = 2,708 Qataris are provided for download in Supplementary Table 1 and online at the Qatar Genome Browser (http://qchip.biohpc.cornell.edu). Shown is a summary of the population enrichment of these variants. **A** Enrichment of potentially pathogenic variants on QChip1 in Qatari subpopulations. In order to determine if Mendelian disease risk alleles were enriched in single Qatari subpopulations, a cross-population allele frequency comparison was conducted for five ancestries observed in Qatar (k1, QGP_PAR, Peninsular Arabs; k2, QGP_GAR, General Arabs; k4, QGP_WEP, Arabs of Western Eurasia and Persia; k5, QGP_SAS, South Asian Arabs, and k3, QGP_AFR, African Arabs). Not shown, QGP_ADM, Admixed Arabs. For each subpopulation, the risk allele frequency was compared to the maximum of the other four subpopulations. Shown is the proportion that was highest in the subpopulation for (left-to-right) QGP_PAR, QGP_GAR, QGP_WEP, QGP_SAS, and QGP_AFR. **B** Enrichment of potentially pathogenic variants on QChip1 in the Qatari genome relative to non-Qatari. The non-Qatari genomes were residents of New York City (total *n* = 226) and Puerto Rico (*n* = 51). The ancestry proportions of these 226 non-Qatari genomes in 5 clusters (k1 to k5) were calculated as described in Fig. 2 (combined analysis of non-Qataris and Qataris using ADMIXTURE^68^), the lowest cross-validation error was for *k* = 5, with the non-Qataris falling in 3 clusters (African-Americans from NYC, *n* = 60, k3; European-Americans from NYC, *n* = 153, k4; South Asian-Americans from NYC, *n* = 13, k5; Puerto Ricans of European Ancestry, k4; and Puerto Ricans of Afro-Caribbean Ancestry, k3). More details of the population structure were made available in Fig. 2 (Qataris) and Supplementary Fig. 1 (non-Qataris). Shown is the percentage of *n* = 32,674 potentially pathogenic variants in Mendelian (single gene) disorder genes that were observed in at least one Qatari and have a risk (minor) allele frequency in Qatar higher than in non-Qatari populations. The proportion of variants was calculated that were at elevated minor allele frequency (enriched) in the Qatari genome relative to the genomes of the 5 non-Qatari population clusters tested: USA African-American (k3), USA European-American (k4), USA South-Asian American (k5), PR Afro-Caribbean (k3), PR European (k4). Shown from left-to-right is the proportion that are enriched in Qatar relative to the maximum of all 5 populations, followed the proportion enriched relative to each individual population.

The applicability of the QChip1 was assessed across populations, including those directly genotyped using the array and others not genotyped in the array but of relevant Greater Middle Eastern ancestry. Of the 32,674 variants of interest for SGD research and screening were observed by QChip1 in at least 1 Qatari, 77% were at a frequency higher than any of the non-Qatari populations genotyped on the array (Fig. 1A). Among the Qatari genomes, the highest proportion of SGD risk alleles were in the Arabs of Western Eurasia and Persia, and African Arab subpopulations (Fig. 1A). As predicted, the majority (76%) of the Qatari genome pathogenic variants were not present in non-Qatari populations (Fig. 1B). QChip1 assessment of NYC and Puerto Rico residents demonstrated only rare detection of Qatari pathogenic variants in populations that included (based on genetic analysis of population clusters, Supplementary Fig. 1) European-American, South Asian-American, African-American populations (Table 5, Supplementary Table 3).

Within the subset of the variants that are known pathogenic and of interest for screening (*n* = 140), similar results were observed for Western populations, with only 6% of QChip1 pathogenic variants observed in Puerto Rico and only 13% found in NYC. Within Arab populations, the results were better but still not sufficient to justify the use of the array, with only 24% of QChip1 pathogenic variants observed in Kuwait and 15% reported in the Center for Arab Genetics Studies database.

### Array performance

Using NGS data as the gold standard, the authors calculated the analytical sensitivity, specificity, accuracy, positive predictive value, and negative predictive value of QChip1. Using data from WGS and QChip1 for *n* = 140 (mostly rare) pathogenic variants in *n* = 472 Qatari, comparison was conducted for *n* = 66,220 genotypes. Of these, *n* = 39,286 could not be compared due to missing genotype in one of the two platforms, (99.8% were missing in WGS only), and among the remaining *n* = 26,934 there were *n* = 26,781 true negatives, *n* = 132 true positives, *n* = 21 false negatives, and *n* = 0 false positives. Based on these data, the sensitivity was 86.3%, the specificity was 100%, the accuracy was 99.9%, the positive predictive value was 100%, and the negative predictive value was 99.9%. This performance is very high relative to recently published evaluations of SNP chips performance on rare pathogenic variants^33^.

## Discussion

This report described the design, testing, and application of QChip1, the first genotyping microarray specifically designed for precision medicine in the Greater Middle Eastern population. QChip was designed for and determined to be suitable for SGD research, clinical screening of newborns or couples planning children, and for genetic diagnosis of SGD patients in the country and in the region.

The main hypothesis of this project was confirmed, that variants of interest for SGD pathogenicity research and screening within known genes vary considerably across populations, as the majority of the QChip1 variants observed in Qatar were either Qatar-private or Qatar-enriched, and were absent from other GME populations and databases of SGD pathogenic variants specific to GME populations. In addition, the majority of QChip1 variants were absent from the Thermo Fisher database, one of the largest knowledgebases in the world of genetic disease variants used in clinical genetics and research genetics. Given the low cost (<$100 each array) and ease of use of the QChip1, it provides an accessible and sustainable alternative to extensive sequencing and interpretation of variants of unknown significance^34^ for the implementation of precision medicine in countries such as Qatar.

The development of QChip1 included the following steps: (1) assessment of the Qatari population to identify Qatari variants and genes of interest for SGD pathogenicity research and screening; (2) design and manufacture of genotyping probesets for inclusion in the QChip1 microarray; (3) refinement and testing of QChip1 by analysis of data from 469 Qataris also sequenced using WGS; and (4) use of the refined QChip1 for quantification of variants of interest for SGD pathogenicity research and screening in 2708 Qatari genomes, with a focus on (a) variants specific-to or enriched-in Qatar relative to non-Qatari DNA samples also genotyped using QChip1 and (b) variants known to be pathogenic.

The key findings of this study were that out of over 104 million variants in Qatar, extensive analysis both in silico and in vitro identified with over 99% accuracy over 32 thousand variants in the Qatari population that are known or predicted to alter the function of genes with a known role in SGDs. The majority of these 32 thousand variants were only observed in Qatar, including 103 of 140 (64%) known pathogenic variants previously observed in Qatari clinical case reports and in ClinVar. Of those variants also observed in Kuwait, the CAGS database of GME variants, NYC or Puerto Rico, the majority were enriched in Qatar, at a higher risk allele frequency. These observations confirm the hypothesis that a considerable proportion of SGD risk variants are population-private founder variants or population-enriched variants that drifted to elevated allele frequency in Qatar. Surprisingly, this hypothesis holds even when compared to neighboring GME populations. This observation justifies the effort invested this research team in developing QChip1 and in producing a framework for the development of similar SGD clinical and research arrays for other understudied populations in the GME, the Americas, and beyond. The population genetic analysis presented here suggests that the high diversity of the Qatari population demonstrates the limited applicability of this array in the Greater Middle East region, which from a genetic perspective spans from Africa to Southern Europe, the Near East, Central Asia, and South Asia. The population-specificity of the variants on the array is a confirmation of the uniqueness and genetic isolation of the Qatari population as previously described by this research team.

The majority of genotyping arrays in use today were designed for coverage of the whole genome, and provide limited coverage of rare variants in genes known and potentially pathogenic in genetic disorders^35^. Screening arrays do exist, most designed for detection of cytogenetic defects in newborns^36^, arrays designed for pre-natal screening^37^, and exome arrays designed for exome-wide association studies (ExWAS)^38^. Exome sequencing is growing in popularity for the detection of risk variants, and a number of companies offer it as a service, including variant interpretation^39^. The challenge with exome sequencing is for clinical use is how to deal with the identification of variants of unknown significance^40^. In contrast, the concept of the QChip1 array is that all variants in the array were annotated prior to genotyping, hence circumventing the issue of variants of unknown significance issues while still covering rare variants. In this sense, the QChip1 knowledgebase is of great value, as it can be used to aid the interpretation of genetic data produced by targeted sequencing or genotyping of a panel of variants of interest for carrier screening, similar to the Plain Insight Panel^41^.

The challenge for array design is the selection of variants. There are over 7 million known missense and loss of function variants^42^, and no array can fit all. Unlike arrays designed for ExWAS, genome-wide association study (GWAS) and population genetics, limiting the array to common variants is not useful for screening for pathogenic variants, as common variants are less likely to be pathogenic, and rare variants are difficult to impute using reference panels and common variant genotype data^43^. In order to focus on pathogenic rare variants, arrays custom-tailored to a population are a better fit for individuals sampled from that population, as rare variants are more likely to be population-specific^44^.

This study provides advances in both knowledge and technology for the field of genomic medicine for a specific genetic population. On the knowledge front, it contains the largest knowledgebase of variants of interest for genetic disease research and screening in a Greater Middle Eastern population. While the consequences of many of the variants on QChip1 are unknown, the array provides a paradigm for clinical screening of this population and a platform for future genetic disease research in the Greater Middle Eastern populations. The variants included in the design and validated in a batch of *n* = 2708 Qatari were as rare as 1 in 5000 (minor allele frequency of 0.0002), and future whole-genome sequencing of Qataris are expected to yield thousands of additional variants of interest. A high confidence in the true existence of such rare SGD risk variants in the Qatari population was boosted by this study, as the variants were discovered by WGS and verified by QChip genotyping.

The QChip1 array did not include short tandem repeats, other repetitive variants, copy number variants, or structural variants. A small proportion of probes on QChip1 were designed for indel detection, but the concordance with whole-genome sequencing for the indels was inadequate. This may be due to inadequate probeset design and should be a focus for future QChip designs. The main limitation of arrays is the space for probes, and in this case the majority of variants were novel to the Axiom platform and hence required multiple probesets. In future iterations, the highest performing probesets identified in this study can be used, and poor performing probesets can be eliminated, thus making additional space on the array for additional variants. Thus, multiple iterations of QChip are needed to produce a high-quality design that genotypes a variety of variants. Another strategy that is frequently used by genotyping array manufacturers is to spread a design across multiple arrays that are genotyped together, i.e., the manufacturers can advertise an array with up to 5 million variants, in reality the “array” consists of 4 or more individual arrays^45^.

Another limitation of this study is *cis/trans* phase of variants, a challenge for exome sequencing. For example, multiple pathogenic variants in BTD can occur in the same genome, and hence screening for these variants includes a second step to determine phase^46^. In the case of this study, there were three pathogenic variants in BTD (rs397514369, rs13078881, rs138818907). Among those individuals with a BTD pathogenic variant, there were five heterozygotes for rs397514369, *n* = 4 homozygotes and *n* = 135 heterozygotes for rs13078881, and *n* = 5 heterozygotes for rs138818907. Zero individuals were positive for more than one BTD pathogenic variant, which rules out the possibility of two pathogenic variants in trans. However, were it the case that multiple BTD variants were observed in the same genome, follow-up validation of phase by Sanger sequencing would be needed. This is a disadvantage of exome sequencing and exome-focused array genotyping, as insufficient coverage of intergenic regions is available for phase inference. Follow-up sequencing is needed, until genome-wide technologies are widely available, such as WGS. Plans for QChip2 include broad coverage of sufficient variants for phase inference.

QChip1 was designed to be competitive relative to sequencing and existing arrays, hence there was a focus on achieving a platform that could provide data for under $100 per DNA sample, including reagents and labor. This is a price point that should remain competitive compared to alternative options for up to a decade, and remains the objective of major manufacturers of sequencing instruments^47^. A major saving is the small data footprint of the QChip1, relative to exome or genome sequencing, where orders of magnitude more data storage are needed. In particular, if the objective is to apply QChip1 on a national scale, the infrastructure investment is considerably more manageable for the prospect of running hundreds of thousands of arrays relative to sequencing hundreds of thousands of genomes or exomes. In perspective, the total Qatari population is approximately 300,000, so the entire Qatari population could be screened for all known and potentially pathogenic variants for approximately $30 million. As presented by the chair of the Qatar Foundation, HH Sheikha Moza bin Nassert at the WISH 2018 summit in Doha, such a precision medicine objective is under consideration for the next decade^48^.

Assessment of 2708 Qatari genomes shed novel insight into the Qatari population. As predicted from our prior assessments of the Qatari population^3,11^, the majority of the pathogenic and predicted pathogenic variants were Qatari-specific, underrepresented in non-Greater Middle Eastern genomes. The most commonly known and high predicted severity pathogenic variants were structural interaction variants and stop gain loss-of-function variants. The most pathogenic variants per genome were observed in the General Arab population, a finding that has implications for other Greater Middle East populations such as Kuwait, United Arab Emirates, and Saudi Arabia that share considerable ancestry with Qatar^18,49–51^. The median Qatari genome had 134 known or computationally predicted pathogenic alleles of interest for SGD research or screening. Of the known pathogenic alleles that were both previously observed in Qatar and known to the ClinVar database, the most common known pathogenic variants were causative of biotinidase deficiency, Stargardt disease, and homocystinuria. Among these 3 variants with risk allele frequency above 0.5% in Qatar, one was not previously known to the CAGS nor HMC databases NM_000060.2(*BTD*):c.[470G > A;1330G > C] linked to biotinidase deficiency. This is unusual, given the high frequency of the pathogenic variant at 0.0265, and could be an indication that either biotinidase deficiency is under-diagnosed in Qatar, or that the variant should be re-classified as “uncertain significance”. The other two variants with elevated risk allele frequency, one was reported in CAGS but not HMC database, NM_000350.2(*ABCA4*):c.[5512C > G;5882G > A] linked to Stargardt disease, risk allele frequency 0.0207. Again, it is unusual that the variant was not previously observed in the HMC database, although it is a known pathogenic variant in Arabs and quite possibly enriched in a subset of the Qatari population due to drift. The NM_000071.2(*CBS*):c.1006C > T (p.Arg336Cys) variant linked to homocystinuria is a well-known variant that is present in both the HMC and CAGS databases, and is known to be an enriched founder variant in the population. It was notable that this variant was incorrectly annotated by SnpEff as “structural interaction”, and only manual review based on the rsID identified the known function (Arg336Cys). This is an issue with annotation software that is not exclusive to SnpEff, where multiple transcripts overlap a variant (4 in the case of *CBS*), and the annotation for the “canonical” experimentally validated function of the variant in disease is buried among other annotations. This is a general problem in variant annotation, and computationally predicted annotations are to be considered an estimate that needs to be validated both by manual review of the literature and experimental validation in vitro. Other known pathogenic variants found using QChip1 included a Factor XI deficiency variant that was previously observed in both Arabs and in ancestral Jewish populations^52^.

QChip1 was designed to assess for pathogenic variants in SGDs, with the aim of genomic medicine for Qatari newborns, premarital couples and clinical genetics patients. A likely future strategy for QChip2 and beyond will be to produce multiple arrays for different purposes, including (1) genome-wide association array designed for genotyping of common variants and calculation of polygenic risk scores for multifactorial disorders^53^; (2) imputation of rare variants based on a Qatari genome imputation reference; (3) population-specific variants that influence drug kinetics and adverse effects; (4) structural variants and repeats; (5) expansion of the QChip1 SGD variants based on a larger sample of Qatari genomes; and (6) variants relevant to autoimmune disease and infectious disease in HLA^54^ and non-autosomal chromosomes, such as ChrX variants in the *ACE2* receptor used by the SARS-Cov-2 virus to infect human cells^55^.

In addition to future versions of the array, the QChip knowledgebase and browser (Qatar Genome Browser) will continue to expand and be updated as more public data from Qatar and literature data on known SGD variants and genes become available. The knowledgebase, array, and browser produced by this project were intended as a first and enabling step towards advancing the state of the art of genomic medicine in Qatar and in populations that share ancestry with Qatar, as demonstrated in the population genetics analysis presented in this study. The intent is to demonstrate this approach as a framework for the development of precision medicine in populations of countries in continents such as Africa^56^, where a per-sample genome analysis cost beyond $100 is out of reach. Given the low cost of sequencing data production, the availability of cloud-based genome analysis infrastructure that does not require large capital investment, and the ease of rapid array design using the Axiom platform, a nation or population that currently has no prior knowledge of genetic variation could take the approach presented here and produce a genetic disease screening program in under a year, potentially saving thousands of lives at risk of unknowingly being affected by a genetic disorder.

The applicability of the QChip1 technology in the Qatari national population is clear, as all of the variants genotyped were previously observed in Qatari nationals, and we know from current and prior studies that the Qatari population sample used as the source of genetic variation for the QChip is also very diverse, with contributions of ancestry from Africa, Europe, and Asia^11,12^. The applicability to expatriates both living within Qatar and those outside of Qatar will depend on shared ancestry between the expatriate individual and the Qatari population. An expatriate coming from one of the populations that contribute to Qatari ancestry will be more likely to have one or more pathogenic variants in QChip. More distantly related individuals would see less benefit from QChip for screening. Confirming that hypothesis, only 6% of the known pathogenic variants were observed in Puerto Ricans, hence an expatriate from Puerto Rico in Qatar would not benefit as much from QChip1 screening as an expatriate from Kuwait, where 20% of QChip1 pathogenic variants were observed. Across the Greater Middle East region, a total of 50% of the QChip1 variants were observed. This study provides a strong argument for ancestry inference as a standard part of precision medicine, to determine the appropriate screening tool and allele frequency reference database for SGDs.

## Methods

### Subject recruitment and sample collection

All research participants were recruited using IRB-approved protocols and informed consent. Recruitment sites included Doha, Qatar (Weill Cornell Medicine – Qatar Institutional Review Board); New York, New York, USA (Weill Cornell Medicine Institutional Review Board); and Mayaguez, Puerto Rico, USA (Institutional Review Board, University of Puerto Rico at Mayagüez). Every research participant received and understood the accurate information in the consent document and other written information and (s)he released the permission to take part in the research by signing the informed consent. No plan was put in place for recontacting participants with information on actionable findings. DNA extracted from whole blood^57^ was tested for quality by RUCDR Infinite Biologics (Piscataway, New Jersey) to be of sufficient quality for array genotyping^58^.

### Strategy to design and assess QChip1

**Fig. 2:**
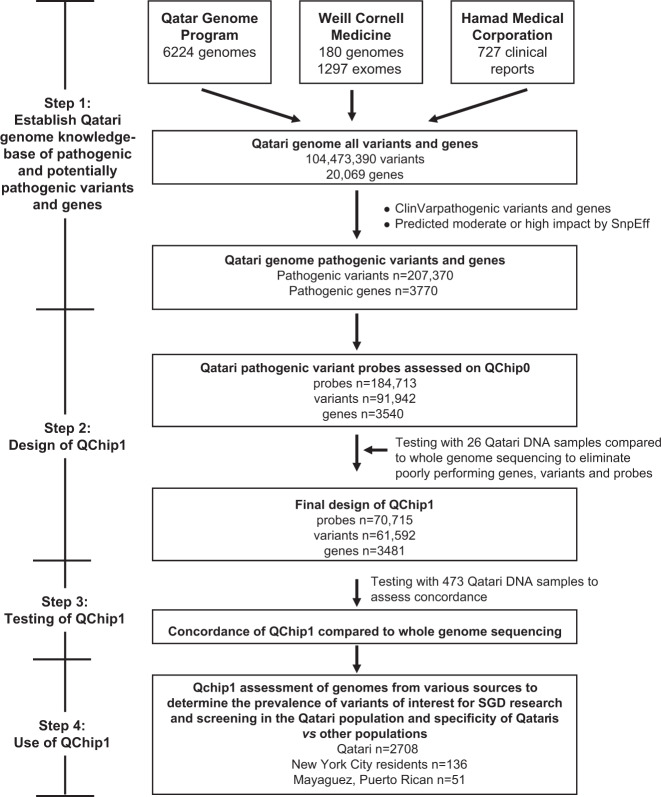
Strategy to design and assess QChip1. Step 1. Qatari Genome Knowledgebase. Identification of the single gene (Mendelian) pathogenic variants and genes in protein coding regions of the Qatari genome was generated using whole-genome sequencing, exome sequencing and clinical reports (see Table 1). After cataloging all variants and respective genes, the pathogenic variants and genes were identified using ClinVar and SnpEff. Step 2. Using this list, Qchip0 (the precursor of QChip1) was designed on the Axiom platform which was then tested with 25 Qatari DNA samples for which whole-genome sequencing was available. Step 3. Elimination of poor performance probes and variants led to the final design of QChip1, which was tested for concordance with genome sequencing using DNA samples from Qataris. Step 4. Use of QChip1 to assess the prevalence of pathogenic variants and genes among Qataris, New York City residents and Puerto Ricans.

QChip1 was developed in steps (Fig. 2). Step 1. Pathogenic variants (known and predicted) in the coding regions of single genes in the Qatari genome were cataloged. Step 2. Using these data, QChip0 (the precursor of QChip1) was designed on the Axiom platform, tested using Qatari genomes and refined with optimal probes, variants and genes to create QChip1. Step 3. QChip1 was tested for concordance with whole-genome sequencing. Step 4. QChip1 was used to evaluate pathogenic variant Qatari prevalence and specificity by assessing genomes from Qataris and non-Qatari populations.

### Step 1: Identification of variants of interest for research or screening in the Qatari Genome

The knowledgebase of pathogenic variants in the Qatari genome was established from several sources, including (1) Qatar Genome Program whole-genome sequencing of 6218 Qatari genomes sequenced on the Illumina HiSeq (Illumina, San Diego, CA) at Sidra Medicine (Doha, Qatar); (2) Department of Genetic Medicine, Weill Cornell Medicine whole-genome sequencing of *n* = 180 Qatari genomes sequenced on the HiSeq at Illumina (*n* = 108)^12^ and the New York Genome Center (*n* = 72)^26^; (3) exome sequencing of *n* = 1297 Qatari genomes sequenced on the HiSeq at Beijing Genomics Institute (*n* = 100)^3^ or New York Genome Center (*n* = 1197)^11^; and (4) *n* = 594 variants from *n* = 721 case reports of hereditary disorders identified by the Clinical Genetics Laboratory at Hamad Medical Corporation (HMC; Doha, Qatar; Supplementary Table 1). The HMC variants were collected in the period between 2002 and 2017, all probands were Qatari nationals. Details of the number of variants in each cohort were tabulated. The final knowledgebase without duplicates consisted of *n* = 104,473,390 variants, including single nucleotide variants (SNVs) and indels (short insertions and deletions; Table 1)

The identification of variants of interest for SGD research and screening in the Qatari genome was carried out in a 3 step process: (1) establishing a list of genes with a known link to Mendelian SGDs described in the ClinVar (version 7/21/20) database; (2) identification of Qatari variants computationally predicted to alter the function of SGD genes in a pathogenic maner, which are primarily of interest for SGD pathogenicity research, and (2) identification of Qatari variants known to be pathogenic in SGDs, based on being classified as such by the ClinVar database or by the HMC case reports.

#### Establishing a list of genes

A list of genes was compiled from ClinVar with the following criteria: (i) protein coding gene in human genome that (ii) has a known link to a SGD and (iii) contains one or more variants in ClinVar that are classified with a “clinical significance” value of “pathogenic” (Supplementary Table 2), recommended by American College of Medical Genetics (ACMG) for variants interpreted for Mendelian disorders^59^.

#### Identification of variants of interest for SGD pathogenicity research in Qataris

Single nucleotide variants (SNV) and indel variants in the Qatar Genome Knowledgebase were annotated using data from public and private sources. First, the allele frequency for each variant in Qataris and non-Qataris was calculated. Variants with a minor allele frequency above 5% in either Qataris or non-Qataris were excluded, per ACMG guidelines^59^. Second, variants were annotated with respect to impact on protein-coding genes in the ENSEMBL database^60^ using SnpEff^61^. Variants that did not affect the function of a SGD gene from ClinVar identified as described above were excluded. Third, variants that were predicted to produce missense or loss-of-function (LoF) variants were kept: these variants are classified by SnpEff as having “High” or “Moderate” potential impact on protein function. This collection of variants includes a variety of variants, including known pathogenic variants, variants of unknown significance, and benign variants.

#### Identification of pathogenic variants for SGD screening

Among the variants defined in step 1.2, a subset is known pathogenic variants, including those classified by ClinVar as pathogenic or those previously observed in HMC case reports of SGDs. These variants can be used for screening of Qataris in a Precision Medicine setting.

### Step 2: Design of QChip1

The microarray platform for the QChip was based on the Axiom custom array platform capable of accommodating 1.3 × 10^6^ probe features, each consisting of DNA probes covalently linked to a silicon wafer designed to hybridize DNA for the genomic sample. Multiple probes designed to hybridize to a genomic segment can be included in a single “probeset”, and one or more probesets designed to genotype a single variant can be included in the design, such that the performance of probes sets can be compared. The initial design was named “QChip0” and the final (post-quality-filtering) version as “QChip1”. The array design contained 693,652 probes in 597,049 probesets. A subset of *n* = 184,713 of the probes (27%), the focus of this report, were designed to assess variants of interest for SGD pathogenicity research and screening. These variants are computationally predicted or are known to affect the function of ClinVar SGD genes found in the variant knowledgebase. The remaining 73% of probes on QChip0, not the subject of this report, were designed for research purposes focused on population genetics, pharmacogenomics, and multifactorial disease research, and will be described in future publications based on future versions of QChip.

The probesets included probes complementary to reference and variant alleles, plus flanking sequence of 35 bases in both 5’ and 3’ directions. Note that this manuscript refers to reference GRCh38 and variant alleles from a genome sequencing perspective. However, in microarray genotyping, there is no “reference” allele, as both alleles are treated as equal by the technology, and hence potentially reducing false genotype calls attributable to reference bias^62^. Some variants were already present in the ThermoFisher (previously Affymetrix) knowledgebase, and thus previously validated to provide accurate genotypes for an SNV or indel, were assessed using a single probeset, while novel variants were assayed using two or more probesets.

Once the array was manufactured, it was tested on an initial batch of genomic DNA samples, including *n* = 26 Qataris from the Weill Cornell Medicine cohort WGS data. Genotypes were generated from the WGS data for these *n* = 26 using GATK Haplotype Caller 3.8^63,64^, configured to output genotypes for all sites on the QChip list, including homozygous reference calls. Comparison of QChip and WGS genotypes was conducted for sites where both WGS and QChip produced a non-missing (sufficient quality) genotype.

In order to exclude poorly performing probesets, two rounds of filtering were applied, including a primary filter to select the highest performing probeset for each variant with multiple probesets, and a secondary filter to exclude variants with a high rate (>10%) of missing genotypes or high rate of discordant genotypes. Excluding poorly performing probes and variants led to the final design of QChip1 with 166,695 probes designed to detect 83,542 variants of 3438 genes. Concordance and filtering analysis were performed using Python^65^ scripts. The concordance analysis script takes as input two single-sample VCF files^66^ as input, including one with QChip1 genotypes and a second with WGS genotypes for all QChip1 sites (including reference and variant genotypes) by GATK 3.8^64^.

### Step 3: Test of QChip1

The concordance of genes and variants of QChip1 with whole-genome sequencing data was calculated for a second array genotyping batch of *n* = 443 Qatari genomic DNA samples previously sequenced using WGS by the Qatar Genome Program. Concordance was performed using the same method for the first batch of *n* = 26 as described above.

### Step 4: Use of QChip1

**Fig. 3:**
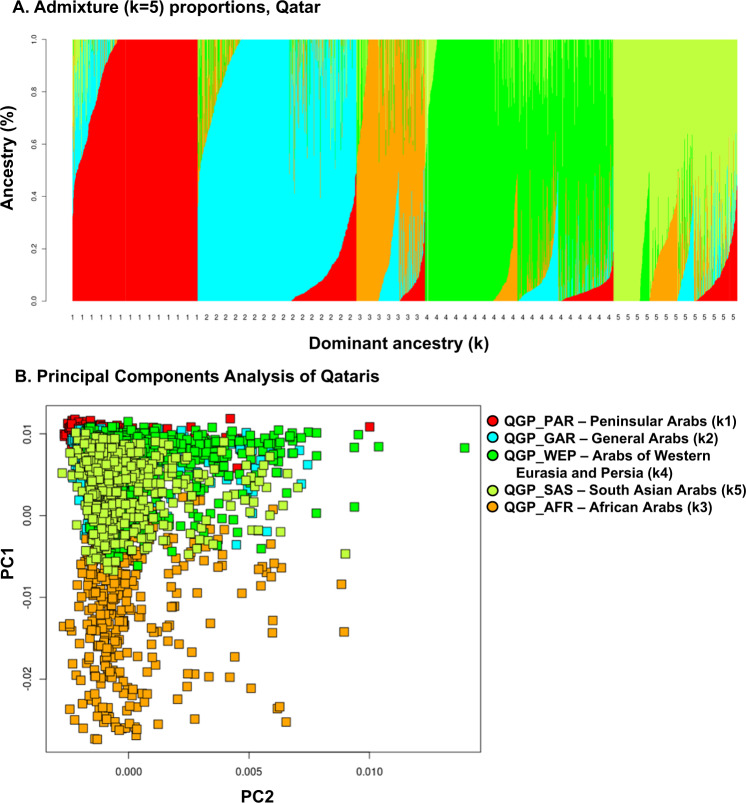
Population structure and principal component analysis of ancestry assessed by QChip1. Sites and samples that failed QC based on variant batch effects or PC outliers were excluded. After QC, ADMIXTURE analysis was conducted on the remaining *n* = 37,674 variants and *n* = 2985 samples of Qataris (*n* = 2708) and non-Qataris (*n* = 277) for a range of K from 3 to 12. The lowest cross-validation error was observed for *k* = 5 for the full dataset. After analysis, the Qatari and non-Qatari samples were plotted separately, the panels here show the Qatari samples from the joint analysis. **A** Admixture (*k* = 5) proportions. Shown is a plot of the admixture proportions (% *k* from 0 to 100%, *y* axis), with each column representing one genome, sorted from left-to-right by dominant (highest %) *k*, and decreasing % k1 to k5. Genomes are color-coded by the dominant (largest %) ancestry (QGP_PAR, Peninsular Arabs, red; QGP_GAR, General Arabs, orange; QGP_WEP, Arabs of West Eurasia and Persia, bright green; QGP_SAS, South Asian Arabs, olive green; and QGP_AFR, African Arabs, light blue). Samples from prior studies of Qatar population structure (Qatar Genome public samples from Fakhro et al.^11^ and Rodriguez-Flores et al.^12^ genotyped on QChip1 were included in the clustering analysis and were used to assign the clusters. **B** Principal components analysis of Qataris. Shown is a PC1 × PC2 plot of Qatari genomes in squares color-coded by cluster of largest proportion of inferred ancestry. Not shown, QGP_ADM, Admixed Arabs.

QChip1 was then used to determine the prevalence of variants of interest for SGD research and screening in the Qatari population (*n* = 2708) compared to genomes for European-American, South Asian-American and African-American New York City (NYC) residents (*n* = 226) and European and Afro-Caribbean in Puerto Rico (PR) residents (*n* = 51). In addition to assessment of variant prevalence in Qataris as a single population, the population structure of Qataris was quantified as described previously^67^, and the prevalence of each variant was quantified for each known Qatari population cluster [Peninsular Arab (QGP_PAR), General Arab (QGP_GAR), Admixed Arab (QGP_ADM), Arabs of Western Eurasia and Persia (QGP_WEP), South Asian Arabs (QGP_SAS) and African Arabs (QGP_AFR); this nomenclature has replaced our prior nomenclature for these subgroups of Q1a, Q1b, Admixed, Q2a, Q2B and Q3, respectively, used in prior publications; Fig. 3]^11^. The population structure was quantified using ADMIXTURE^68^ for both Qataris and non-Qataris (Supplementary Fig. 1) using QChip1 data that was filtered to exclude indels, singletons, and variants in linkage disequilibrium (window 1000, step 25, maximum *r*^2^ 0.1). Each genome was assigned to an inferred population cluster based on the *k* value with lowest cross-validation error (*k* = 5). Rather than classify individuals as admixed/non-admixed, each individual genome was assigned to the cluster (k) with the highest proportion of ancestry^69^. The results were visualized in a plot of principal components (PCs) calculated using PLINK^70^, with visualization in R^71^. Outliers were excluded based on over 2 standard deviations outside the median PC value for PCs 1 to 5. Each genome was color-coded by the inferred ancestry (1–5) and the country of origin (Qatar, US, PR).

### Data analysis

The final set of QChip1 data included SNV variants with high-quality genotypes and genomes with known ancestry that are of interest for research and screening of SGDs in Qataris. Analysis of these data included quantification and comparison across populations of the following parameters: (1) individual burden of variants; (2) prevalence of variants; (3) enrichment of variants among Qatari subpopulations; and (4) enrichment of variants in Qataris compared to non-Qatari populations.

### Performance

Once a final set of pathogenic variants screened using QChip1 was identified, the performance of the array was quantified. Data for QChip1 and WGS was compared on *n* = 140 pathogenic variants for *n* = 472 genomes. Using WGS as a “gold standard”, the number of true negative (TN, both WGS and QChip1 call wild type genotype), true positive (TP, both WGS and QChip1 call heterozygote or homozygote for risk allele), false negative (FN, WGS calls positive but QChip1 calls negative), and false positive (FP, WGS calls negative and QChip1 calls positive). Based on these four numbers, the sensitivity [TP/(TP + FN)], specificity [TN/(TN + FP)], accuracy [TP/(TN + TP + FN + FP)], positive predictive value [TP/(TP + FP)], and negative predictive value [TN/(TN + FN)] was calculated.

### Utility beyond Qatar

In order to assess the potential utility of QChip1 beyond Qatar, the number of QChip1 pathogenic variants was quantified in internal and external knowledgebases. The internal knowledgebases included the QChip1 data for Qatar, NYC, Puerto Rico, and the Hamad Medical Corporation (https://www.hamad.qa/EN/Pages/default.aspx) list of pathogenic variants. The external knowledgebases included ClinVar (https://www.ncbi.nlm.nih.gov/clinvar/), the Center for Arab Genetics Studies (https://www.cags.org.ae/en), the Iranome (http://www.iranome.ir/), the GME Variome (http://igm.ucsd.edu/gme/), and a set of exomes sequenced by the Dasman Diabetes Institute in Kuwait (https://www.dasmaninstitute.org/). Among the external databases, allele frequency was available for Iran (*n* = 800), GME (*n* = 886), and Kuwait (*n* = 540). The subset of variants present in one or more of the knowledgebases, as well as the subset present in one or more external knowledgebase focusing on the Greater Middle East region (CAGS, Iran, GME, Kuwait) was also quantified.

### QChip genome browser

In order to provide researchers and clinicians access to annotation and allele frequency data in Qatar and USA for the QChip1 Qatar SGD pathogenicity research and screening variants and genes, a web browser was constructed. The Qatar Genome Browser architecture consisted of a searchable table with a user interface implemented in a Shiny RStudio^72^ application frontend, running within a Docker (docker.com) container instance installed on a Linux Centos (centos.org) server backend. The server was custom built by Red Barn (thinkredbarn.com) and configured by Cornell BioHPC^73^. In order to maintain security, the development version was accessible only within Cornell campus network or via Cornell VPN, with plans for a public release after publication of this report. Testing of the server was conducted to confirm that the url (http://qchip.biohpc.cornell.edu) was accessible from both Weill Cornell Medicine New York and Weill Cornell Medicine Qatar.

### Reporting summary

Further information on research design is available in the Nature Research Reporting Summary linked to this article.

## Supplementary information

Supplementary Information

Reporting Summary

## Data Availability

Public datasets not produced by the authors and used in this study that describe disease genes, variants in disease genes, and their prevalence in Greater Middle East populations are available from ClinVar (https://www.ncbi.nlm.nih.gov/clinvar/), the Center for Arab Genetics Studies (https://www.cags.org.ae/en), the Iranome (http://www.iranome.ir/), the GME Variome (http://igm.ucsd.edu/gme/), and the Thanaraj Lab at the Dasman Diabetes Institute in Kuwait (https://research.dasmaninstitute.org/en/persons/alphonse-thangavel-thanaraj). The data produced by the authors and used in this study can be divided into three categories: (1) sequence and genotype data used to produce the QChip knowledgebase of variants (2) QChip genotype data, and (3) summaries of variants in QChip. For the sake of scientific reproducibility, availability and access to these three categories of data is described here. Category 1 data includes WGS data produced either by the Qatar Genome Program (QGP), Qatar BioBank (QBB) or by Weill Cornell Medicine, WES data produced by Weill Cornell Medicine (WCM), and a table of pathogenic variants previously observed at Hamad Medical Corporation (HMC). The QGP/QBB WGS data is described in Mbarek et al^24^, sharing of these data outside of Qatar is prohibited and is not consented by the IRB protocol. However, external access to QBB/QGP genotype and phenotype data can be obtained through an established ISO-certified process by submitting a project request at https://www.qatarbiobank.org.qa/research/how-apply which is subject to approval by the QBB IRB committee. A detailed description of the data management infrastructure for QBB was described previously^22^. The data and biosamples collected or generated by QBB are available to researchers at public and private institutions that conduct scientific research and that meet the requirements detailed in the Qatar Biobank Research Access policy. Approved Users are given access to QBB’s Research Data and/or Biosamples for the period agreed upon in the approved Access Agreement, with the possibility of subsequent renewal.” For more information on what meets the requirements, researchers can request the Qatar Biobank Research Access policy from qbbrpsupport@qf.org.qa. This policy has enabled data sharing and collaboration in multiple studies, including a population genetics analysis of over 6000 Qataris^25^ and the latest results of the COVID-19 Host Genetics Initiative^74^. Category 1 data also includes WGS and WES data produced by Weill Cornell Medicine, these data are available for sharing with researchers. The majority of these data was described in prior publications and is available for download from NCBI SRA, see SRP060765 for published WGS data, SRP061943 and SRP061463 for published WES data. Unpublished WGS data from this study is accessible Unpublished WGS data from this study is accessible through NCBI BioProject PRJNA774497. Category 1 data also includes an unpublished list of variants identified by HMC, these data are available from a FigShare repository created for this project (https://figshare.com/projects/QChip1/120108). Category 2 data consists of QChip array genotypes for Qataris recruited by WCM, Qataris recruited by QBB, New Yorkers recruited by WCM, and Puerto Ricans recruited by UPRM. Consent for data sharing is not possible for Qataris recruited by QBB as well as for Puerto Ricans recruited by UPRM. QChip array genotypes for Qataris and New Yorkers recruited by WCM was deposited at NCBI (project accession PRJNA774497) and is included in the FigShare repository (https://figshare.com/projects/QChip1/120108). Category 3 data consists of summaries of QChip variants, including annotation from Thermo Fisher (Affymetrix) on the QChip contents, annotation produced by the authors on QChip contents including allele frequency, a list of QChip variants of interest for SGD research, and a list of QChip variants of interest for SGD screening. All four datasets are available through the FigShare repository (https://figshare.com/projects/QChip1/120108). A browsable version of the list of variants with allele frequency data is in development and will be available at the project website (http://qchip.biohpc.cornell.edu). Variants of interest for screening in Qatar on QChip1 were deposited to dbSNP in a batch submission, are expected to be a part of dbSNP build 156, and were assigned the following accessions: ssID 2137544269 and ssIDs 5314393773 through 5314393911. The batch submission is available online at https://www.ncbi.nlm.nih.gov/SNP/snp_viewBatch.cgi?sbid=1063269.

## Code availability

Software code consisting of Python, Bash, and R scripts used to produce and analyze the data presented in this manuscript are available through the GitHub https://github.com/juansearch/qchip1 and on the project website http://qchip.biohpc.cornell.edu.
