# Supplementary material for: The QChip1 knowledgebase and microarray for precision medicine in Qatar
Source: NPJ Genom Med. 2022 Jan 19;7:3. doi: 10.1038/s41525-021-00270-0 (PMC8770564; doi:10.1038/s41525-021-00270-0)

Supplemental Methods

All research participants were recruited using IRB-approved protocols and informed consent. Recruitment sites included Doha, Qatar; New York, New York, USA; and Mayaguez, Puerto Rico, USA. DNA extracted from whole blood[1] was tested for quality by RUCDR Infinite Biologics (Piscataway, New Jersey) to be of sufficient quality for array genotyping[2].

Strategy to Design and Assess QChip1

QChip1 was developed in steps (Figure 1). **Step 1.** Pathogenic variants (known and predicted) in the coding regions of single genes in the Qatari genome were cataloged. **Step 2.** Using this data, QChip0 (the precursor of QChip1) was designed on the Axiom platform, tested using Qatari genomes and refined with optimal probes, variants and genes to create QChip1. **Step 3.** QChip1 was tested for concordance with whole genome sequencing. **Step 4.** QChip1 was used to evaluate pathogenic variant Qatari prevalence and specificity by assessing genomes from Qataris and non-Qatari populations.

Step 1: Identification of Pathogenic Variants in the Qatari Genome

The knowledgebase of pathogenic variants in the Qatari genome was established from several sources, including: (1) Qatar Genome Program whole genome sequencing of 6,218 Qatari genomes sequenced on the Illumina HiSeq (Illumina, San Diego, CA) at Sidra Medicine (Doha, Qatar); (2) Department of Genetic Medicine, Weill Cornell Medicine whole genome sequencing of n=180 Qatari genomes sequenced on the HiSeq at Illumina (n=108)[3] and the New York Genome Center (n=72)[4]; (3) exome sequencing of n=1,297 Qatari genomes sequenced on the HiSeq at Beijing Genomics Institute (n=100)[5] or New York Genome Center (n=1,197)[6]; and (4) n=594 variants from n=721 case reports of hereditary disorders identified by the Clinical Genetics Laboratory at Hamad Medical Corporation (HMC; Doha, Qatar; Supplemental Table I). Details of the number of variants in each cohort were tabulated. The final knowledgebase

without duplicates consisted of n=104,473,390 variants, including single nucleotide variants (SNVs) and indels (short insertions and deletions; Table I)

The identification of pathogenic variants in the Qatari genome was carried out in a 2 step process: (1) establishing a list of genes with an known link to Mendelian SGDs described in the ClinVar (version 7/21/20) database; and (2) identification of Qatari variants known or computationally predicted to alter the function of SGD genes in a pathogenic manner.

1. *Establishing a list of genes.* A list of genes was compiled from ClinVar with the following criteria: (*i*) protein coding gene in human genome that (*ii*) has a known link to a SGD and (*iii*) contains one or more variants in ClinVar that are classified with a "clinical significance" value of "pathogenic" (Supplemental Table II), recommended by American College of Medical Genetics (ACMG) for variants interpreted for Mendelian disorders[7].
2. *Identification of variants known or predicted to be pathogenic in Qataris.* Single nucleotide variants (SNV) and indel variants in the Qatar Genome Knowledgebase were annotated using data from public and private sources. First, the allele frequency for each variant in Qataris and non-Qataris was calculated. Variants with a minor allele frequency above 5% in either Qataris or non-Qataris were excluded, per ACMG guidelines[7]. Second, variants were annotated with respect to impact on protein coding genes in the ENSEMBL database[8] using SnpEff[9]. Variants that did not affect the function of a SGD gene from ClinVar identified as described above were excluded. Third, variants that were predicted to produce missense or loss-of-function (LoF) variants were kept: these variants are classified by SnpEff as having "High" or "Moderate" potential impact on protein function.

Step 2: Design of QChip1

The microarray platform for the QChip was based on the Axiom custom array platform capable of accommodating $1.3x10^6$ probe features, each consisting of DNA probes covalently linked to a silicon wafer designed to hybridize DNA for the genomic sample. Multiple probes designed to hybridize to a genomic segment can be included in a single "probeset", and one or more probesets designed to genotype a single variant can be included in the design, such that the performance of probes sets can be compared. The initial design was named "QChip0" and the final (post-quality-filtering) version as "QChip1". The array design contained 693,652 probes in 597,049 probesets. A subset of n=184,713 of the probes (27%), the focus of this report, were designed to assess known or potentially pathogenic variants in ClinVar SGD genes found in the variant knowledgebase. The remaining 73% of probes on QChip0, not the subject of this report, were designed for research purposes focused on population genetics, pharmacogenomics, and multifactorial disease research, and will be described in future publications based on future versions of QChip.

The probesets included probes complementary to reference and variant alleles, plus flanking sequence of 35 bases in both 5' and 3' directions. Note that this manuscript refers to reference grch38 and variant alleles from a genome sequencing perspective. However, in microarray genotyping, there is no "reference" allele, as both alleles are treated as equal by the technology, and hence potentially reducing false genotype calls attributable to reference bias[10]. Some variants were already present in the ThermoFisher (previously Affymetrix) knowledgebase, and thus previously validated to provide accurate genotypes for a SNV or indel, were assessed using a single probeset, while novel variants were assayed using two or more probesets.

Once the array was manufactured, it was tested on an initial batch of genomic DNA

samples, including n=26 Qataris from the Weill Cornell Medicine cohort WGS data. Genotypes were generated from the WGS data for these n=26 using GATK Haplotype Caller 3.8[11,12], configured to output genotypes for all sites on the QChip list, including homozygous reference calls. Comparison of QChip and WGS genotypes was conducted for sites where both WGS and QChip produced a non-missing (sufficient quality) genotype.

In order to exclude poorly performing probesets, two rounds of filtering were applied, including a primary filter to select the highest performing probeset for each variant with multiple probesets, and a secondary filter to exclude variants with a high rate (>10%) of missing genotypes or high rate of discordant genotypes. Excluding poorly performing probes and variants led to the final design of QChip1 with 166,695 probes designed to detect 83,542 variants of 3,438 genes. Concordance and filtering analysis was performed using Python[13] scripts. The concordance analysis script takes as input two single-sample VCF files[14] as input, including one with QChip1 genotypes and a second with WGS genotypes for all QChip1 sites (including reference and variant genotypes) by GATK 3.8[12].

Step 3: Test of QChip1

The concordance of genes and variants of QChip1 with whole genome sequencing data was calculated for a second array genotyping batch of n=443 Qatari genomic DNA samples previously sequenced using WGS by the Qatar Genome Program. Concordance was performed using the same method for the first batch of n=26 as described above.

Step 4: Use of QChip1

QChip1 was then used to determine the prevalence of single gene pathogenic and potentially pathogenic variants and genes in the Qatari population (n=2,708) compared to genomes for European-American, South Asian-American and African-American New York City (NYC) residents (n=226) and European and Afro-Caribbean in Puerto Rico (PR) residents

(n=51). In addition to assessment of variant prevalence in Qataris as a single population, the population structure of Qataris was quantified as described previously[15], and the prevalence of each variant was quantified for each known Qatari population cluster [Peninsular Arab (QGP_PAR), General Arab (QGP_GAR), Admixed Arab (QGP_ADM), Arabs of Western Eurasia and Persia (QGP_WEP), South Asian Arabs (QGP_SAS) and African Arabs (QGP_AFR); this nomenclature has replaced our prior nomenclature for these subgroups of Q1a, Q1b, Admixed, Q2a, Q2B and Q3, respectively, used in prior publications; Figure 2][6]. The population structure was quantified using ADMIXTURE[16] for both Qataris and non-Qataris (Supplemental Figure 1) using QChip1 data that was filtered to exclude indels, singletons, and variants in linkage disequilibrium (window 1000, step 25, maximum r^2 0.1). Each genome was assigned to an inferred population cluster based on the *k* value with lowest cross-validation error (k=5). Rather than classify individuals as admixed / non-admixed, each individual genome was assigned to the cluster (k) with the highest proportion of ancestry[17]. The results were visualized in a plot of principal components (PCs) calculated using PLINK[18], with visualization in R[19]. Outliers were excluded based on over 2 standard deviations outside the median PC value for PCs 1 to 5. Each genome was color-coded by the inferred ancestry (1 to 5) and the country of origin (Qatar, US, PR).

Data analysis. The final set of QChip1 data included SNV variants with high-quality genotypes and genomes with known ancestry. Analysis of this data included quantification and comparison across populations of the following parameters: (1) individual burden of known and potential pathogenic variants identified using QChip1; (2) prevalence of variants known to be pathogenic in SGDs; (3) enrichment of potentially pathogenic variants among Qatari sub-populations; and (4) enrichment of potentially pathogenic variants in Qataris compared to non-Qatari populations.

QChip Genome Browser

In order to provide researchers and clinicians access to annotation and allele frequency data in Qatar and USA for the QChip1 Qatar pathogenic and potentially pathogenic variants and genes, a web browser was constructed. The Qatar Genome Browser architecture consisted of a searchable table with a user interface implemented in a Shiny RStudio[20] application frontend, running within a Docker (docker.com) container instance installed on a Linux Centos (centos.org) server backend. The server was custom built by Red Barn (thinkredbarn.com) and configured by Cornell BioHPC[21]. In order to maintain security, the development version was accessible only within Cornell campus network or via Cornell VPN, with plans for a public release upon publication of this report. Testing of the server was conducted to confirm that the url (qchip.biohpc.cornell.edu) was accessible from both Weill Cornell Medicine New York and Weill Cornell Medicine Qatar.

Data availability. Processed data is available on the website qchip.biohpc.cornell.edu and raw data is available upon request.

Supplemental References

1. Lauro, F.M., Chastain, R.A., Blankenship, L.E., Yayanos, A.A., and Bartlett, D.H. (2007). The unique 16S rRNA genes of piezophiles reflect both phylogeny and adaptation. *Applied and environmental microbiology* **73**, 838-845.
2. Guha, P., Das, A., Dutta, S., and Chaudhuri, T.K. (2018). A rapid and efficient DNA extraction protocol from fresh and frozen human blood samples. *Journal of clinical laboratory analysis* **32**.
3. Rodriguez-Flores, J.L., Fakhro, K., Agosto-Perez, F., Ramstetter, M.D., Arbiza, L., Vincent, T.L., Robay, A., Malek, J.A., Suhre, K., Chouchane, L., et al. (2016). Indigenous Arabs are descendants of the earliest split from ancient Eurasian populations. *Genome Res* **26**, 151-162.
4. Rodriguez-Flores, J.L., Ramstetter, M.D., Staudt, M.R., Robay, A., Fakhro, K.A., Mezey, J.G., Salit, J., Malek, J., Abi Khalil, C., and Crystal, R.G. (2016). Bioinformatics workflow for whole genome sequence linkage analysis of multiple families afflicted with rare disease of unknown heredity and penetrance. In American Society of Human Genetics 66th Annual Meeting. (Vancouver, Canada, October 18–22, 2016
5. Rodriguez-Flores, J.L., Fakhro, K., Hackett, N.R., Salit, J., Fuller, J., Agosto-Perez, F., Gharbiah, M., Malek, J.A., Zirie, M., Jayyousi, A., et al. (2014). Exome sequencing identifies potential risk variants for Mendelian disorders at high prevalence in Qatar. *Hum Mutat* **35**, 105-116.
6. Fakhro, K.A., Staudt, M.R., Ramstetter, M.D., Robay, A., Malek, J.A., Badii, R., Al-Marri, A.A., Abi Khalil, C., Al-Shakaki, A., Chidiac, O., et al. (2016). The Qatar genome: a population-specific tool for precision medicine in the Middle East. *Hum Genome Var* **3**, 16016.
7. Richards, S., Aziz, N., Bale, S., Bick, D., Das, S., Gastier-Foster, J., Grody, W.W., Hegde, M., Lyon, E., Spector, E., et al. (2015). Standards and guidelines for the interpretation of sequence variants: a joint consensus recommendation of the American College of Medical Genetics and Genomics and the Association for Molecular Pathology. *Genet Med* **17**, 405-424.
8. Zerbino, D.R., Achuthan, P., Akanni, W., Amode, M.R., Barrell, D., Bhai, J., Billis, K., Cummins, C., Gall, A., Girón, C.G., et al. (2018). Ensembl 2018. *Nucleic Acids Res* **46**, D754-d761.
9. Cingolani, P., Platts, A., Wang le, L., Coon, M., Nguyen, T., Wang, L., Land, S.J., Lu, X., and Ruden, D.M. (2012). A program for annotating and predicting the effects of single nucleotide polymorphisms, SnpEff: SNPs in the genome of Drosophila melanogaster strain w1118; iso-2; iso-3. *Fly* **6**, 80-92.
10. Martiniano, R., Garrison, E., Jones, E.R., Manica, A., and Durbin, R. (2020). Removing reference bias and improving indel calling in ancient DNA data analysis by mapping to a sequence variation graph. *Genome biology* **21**, 250.
11. McKenna, A., Hanna, M., Banks, E., Sivachenko, A., Cibulskis, K., Kernytsky, A., Garimella, K., Altshuler, D., Gabriel, S., Daly, M., et al. (2010). The Genome Analysis Toolkit: a MapReduce framework for analyzing next-generation DNA sequencing data. *Genome Res* **20**, 1297-1303.
12. Van der Auwera, G.A., Carneiro, M.O., Hartl, C., Poplin, R., Del Angel, G., Levy-Moonshine, A., Jordan, T., Shakir, K., Roazen, D., Thibault, J., et al. (2013). From FastQ data to high confidence variant calls: the Genome Analysis Toolkit best practices pipeline. *Current protocols in bioinformatics* **43**, 11.10.11-11.10.33.

13. python.org. (2020). The Python Language Reference¶. In. (https://docs.python.org/3/reference/.
14. Danecek, P., Auton, A., Abecasis, G., Albers, C.A., Banks, E., DePristo, M.A., Handsaker, R.E., Lunter, G., Marth, G.T., Sherry, S.T., et al. (2011). The variant call format and VCFtools. *Bioinformatics (Oxford, England)* **27**, 2156-2158.
15. O'Beirne, S.L., Salit, J., Rodriguez-Flores, J.L., Staudt, M.R., Abi Khalil, C., Fakhro, K.A., Robay, A., Ramstetter, M.D., Malek, J.A., Zirie, M., et al. (2018). Exome sequencing-based identification of novel type 2 diabetes risk allele loci in the Qatari population. *PLoS One* **13**, e0199837.
16. Alexander, D.H., Novembre, J., and Lange, K. (2009). Fast model-based estimation of ancestry in unrelated individuals. *Genome Res* **19**, 1655-1664.
17. O'Beirne, S.L., Salit, J., Rodriguez-Flores, J.L., Staudt, M.R., Abi Khalil, C., Fakhro, K.A., Robay, A., Ramstetter, M.D., Al-Azwani, I.K., Malek, J.A., et al. (2016). Type 2 Diabetes Risk Allele Loci in the Qatari Population. *PLoS One* **11**, e0156834.
18. Chang, C.C., Chow, C.C., Tellier, L.C., Vattikuti, S., Purcell, S.M., and Lee, J.J. (2015). Second-generation PLINK: rising to the challenge of larger and richer datasets. *GigaScience* **4**, 7.
19. Ihaka, R., and Gentleman, R. (1996). R: A Language for Data Analysis and Graphics. *Journal of Computational and Graphical Statistics* **5**, 299-314.
20. Studio, R. (2020). Shiny from R Studio. In. (https://shiny.rstudio.com/.
21. Cornell University, I.o.B. (2017). Bioinformatics Internal Site Home. In. (https://biohpc.cornell.edu/Default.aspx.

Supplemental Table I. Whole Genome and Exome Sequencing of the Qatari Genome Used for Identification of Qatari Variants[1]

Source	Sequencing	Sequencing site	n	n/source
Weill Cornell Medicine	Whole genome	Illumina	108	
		New York Genome Center	72	
		Total		180
	Exome	Beijing Genomics Institute	100	
		New York Genome Center	1,197	
		Total		1,297
Qatari Genome Program	Whole genome	Sidra Medicine	6,224	6,224
Hamad Medical Corporation	Clinical reports	Hamad Medical Corporation	727	727
		Total all sequences		8,428

[1] Shown is the genome and exome data contributing single nucleotide variants and indel variants to the QChip knowledgebase. All genomes and exomes were sequenced using the Illumina HiSeq platform. Shown is the source of sample collection, the sequencing breadth (whole genome or exome), the sequencing facility, and the sample size. The Hamad Medical Corporation data is from clinical reports from the Diagnostic Genomic Division.

Supplemental Table II. Pathogenic Variant Sites and Genes in ClinVar[1]

Category	All	Pathogenic
Variants	718,248	155,494
Genes	12,521	3,975

[1] The ClinVar database was downloaded from NCBI in June of 2020. Shown are the total variant and sites subset restricted to high or moderate impact pathogenic variants. The ClinVar database of high or moderate impact pathogenic variants was matched with the Qatari genome database Supplemental Table I) to identify the pathogenic variants/genes in the ClinVar database (Table I).

Supplemental Table III. List of Known and Potential Pathogenic Variants in ClinVar Mendelian Disease Risk Genes Observed in Qatar using QChip1[1]

Column name	Description
genename	Gene symbol
phenos	Phenotypes linked to pathogenic variant in gene, according to ClinVar
affycytoband	Cytogenetic band (Affymetrix annotation)
cpra	Genomic coordinates in chromosome:position:reference:alternate format
hgvsc	Transcript change in HGVS format
hgvsp	Protein change in HGVS format
annotation	Variant annotation, functional class (according to SnpEff)
annotationimpact	Impact (high or moderate)
cpa1a2	Genomic coordinates in chromosome:position: minor (a1) : major (a2) format
a1a1qtr	Minor (risk) allele homozygotes in Qatar
a1a2qtr	Heterozygotes in Qatar
a2a2qtr	Major (wild type) allele homozygotes in Qatar
mafqtr	Minor allele frequency in Qatar
mafqtr1	MAF in Qatar k1 cluster
mafqtr2	MAF in Qatar k2 cluster
mafqtr3	MAF in Qatar k3 cluster
mafqtr4	MAF in Qatar k4 cluster
mafqtr5	MAF in Qatar k5 cluster
mafusa	MAF in USA
mafusa3	MAF in USA k3 cluster
mafusa4	MAF in USA k4 cluster
mafusa5	MAF in USA k5 cluster
mafpr	MAF in PR
mafpr3	MAF in PR k3 cluster
mafpr4	MAF in PR k4 cluster
affyid	Affymetrix ID
affydbsnprsid	DbSNP ID (provided by Affymetrix)
cvpathvar	Is variant known pathogenic according to ClinVar
hmcpathvar	Is variant known pathogenic according to HMC

[1] This table provides details on the final set of n=32,674 known or potentially pathogenic variants in Mendelian disease risk genes that are of interest for research or screening and were observed in one or more Qataris using QChip1. Shown is a list of columns in the file, which is available for browsing or download in Excel format from http://qchip.biohpc.cornell.edu

Supplemental Table IV. Distribution of Types of Functional Variants Identified by QChip1 the Qatari Genome[1]

Predicted impact severity	Predicted protein change[2]	n	%
High	Structural interaction variant	1,134	3.471
	Stop gained	322	0.985
	Splice donor variant & intron variant	157	0.481
	Splice acceptor variant & intron variant	111	0.340
	Protein-protein contact	41	0.125
	Start lost	40	0.122
	Stop gained & splice region variant	17	0.052
	Stop lost	14	0.043
	Start lost & splice region variant	2	0.006
	Stop lost & splice region variant	1	0.003
Moderate	Missense variant	26,545	81.242
	Sequence feature	3,548	10.859
	Missense variant & splice region variant	742	2.271

[1] Shown is the functional distribution of QChip1 variants of interest for SGD research and screening that were observed in the Qatari genome. From left-to-right is the impact category (high or moderate), the predicted protein change, the number of variants in the impact category observed in one or more Qataris, and the percentage of variants represented by each category.

[2] Listed are the SnpEff categories of predicted result of the pathogenic variant.

Supplemental Figure Legends

Supplemental Figure 1. After QC, ADMIXTURE analysis was conducted on the remaining n=37,674 variants and n=2,985 samples (including Qataris, New Yorkers, and Puerto Ricans) for a range of K from 3 to 12. The lowest cross-validation error was observed for k=5. After admixture analysis, the Qatari (Figure 2) and non-Qatari were plotted separately (this figure). **A.** Admixture (k=5) proportions in US and PR. Shown is a plot of the admixture proportions for non-Qataris genotyped using QChip1 (% k from 0 to 100%, y axis), with each column representing one genome, sorted from left-to-right by dominant (highest %) k, and decreasing % k1 to k5. Genomes are color coded by the dominant (largest %) ancestry. **B.** Principal components analysis of US and PR. Shown is a PC1 x PC2 plot of US and PR genomes genotyped using QChip1 in squares color-coded by cluster of largest proportion of inferred ancestry.

- k4 – European-American in NYC or European-Caribbean in PR
- k5 – South Asian American in NYC
- k3 – African-American in NYC or Afro-Caribbean in PR

A. Admixture (k=5) proportions in US and PR

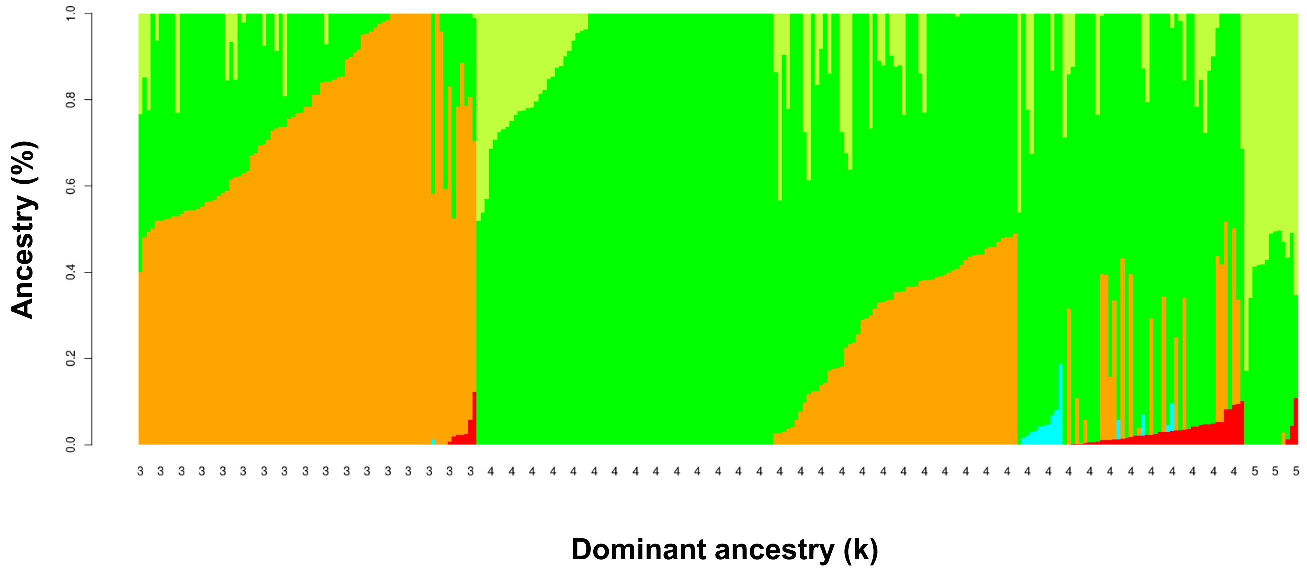

B. Principal components analysis of US and PR

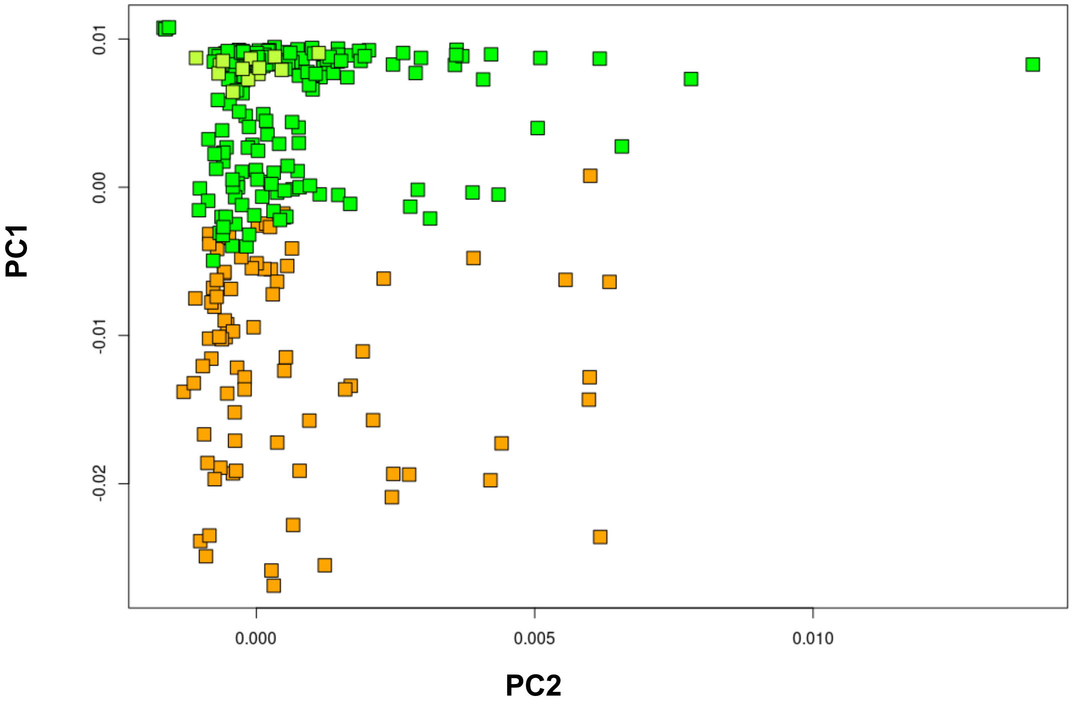

Supplement: Supplementary file 1 — Supplementary Information [file 41525_2021_270_MOESM1_ESM.pdf]
